# MntJULiP and Jutils: differential splicing analysis of RNA-seq data with covariates

**DOI:** 10.1093/nargab/lqaf140

**Published:** 2025-11-03

**Authors:** Wui Wang Lui, Guangyu Yang, Zitong He, Liliana Florea

**Affiliations:** Department of Computer Science, Johns Hopkins University, Baltimore, MD 21205, United States; Department of Computer Science, Johns Hopkins University, Baltimore, MD 21205, United States; META, 101 Burlingame Ave, Burlingame, CA 94010, United States; Department of Computer Science, Johns Hopkins University, Baltimore, MD 21205, United States; Department of Computer Science, Johns Hopkins University, Baltimore, MD 21205, United States; Department of Genetic Medicine, Johns Hopkins School of Medicine, Baltimore, MD 21205, United States

## Abstract

Emerging large and complex RNA-seq datasets from disease and population studies include multiple confounders such as sex, age, ethnicity, and clinical attributes, which demand highly specialized data analysis tools. However, current methods are generally not equipped to handle the new challenges. We describe an extension of our programs MntJULiP and Jutils for differential splicing detection and visualization from RNA-seq data that accounts for covariates. MntJULiP detects intron-level differences in both splicing ratios and splicing abundance from RNA-seq data using a Bayesian linear mixture model adjusted for covariates. Jutils visualizes alternative variation with heatmaps, sashimi plots, Venn diagrams, and, reported here, PCA maps. With covariate modeling, MntJULiP drastically reduces false positives to achieve very high precision (>90%), significantly outperforming competitors. We applied the methods to GTEx brain RNA-seq samples to deconvolute the effects of sex and age at death on the splicing patterns. In particular, analyses of frontal cortex data reveal a pattern of increased splicing differences with more distant age groups, while clustering of covariate-adjusted data identifies a subgroup of individuals undergoing a distinct splicing program over the age span.

## Introduction

Differences in alternative splicing patterns are responsible for the diversity of proteins across tissues, cell types, and developmental stages, and disruptions in normal RNA splicing patterns have been reported in a number of diseases [1, 2]. Increasingly large and complex RNA-seq datasets that include multiple confounders, such as sex, age, ethnicity, and clinical attributes, are emerging from disease study cohorts and population-level projects, which demand highly specialized analysis tools. While multiple methods exist to detect differences in splicing, including LeafCutter, MntJULiP, rMATS, SUPPA2, DRIMSeq, DEXSeq, and DARTS [3–9], few are equipped to handle the complexities of the data. In particular, there is a scarcity of programs that can rigorously account for the effect of confounding attributes on the observed data. Additionally, visualization tools are critical for enabling biologists to quickly and intuitively interpret such differences, identifying patterns genome-wide or at the level of the individual genes. We previously developed two tools, MntJULiP [4] and Jutils [10], for differential splicing detection and visualization, respectively, that can efficiently handle large-scale and complex RNA-seq data collections. We report a recent implementation of these programs to account for covariates and to produce new customizable visualizations of covariate-adjusted data as PCA plots. We demonstrate their accuracy on simulated data. We then illustrate their applicability and usefulness by analyzing RNA-seq data from brain tissue obtained from the GTEx repository [11], which paints the global splicing landscape across age and biological sex groups and identifies a potential distinct splicing program in a subset of individuals.

## Materials and methods

### Overview of the MntJULiP tool

MntJULiP [4] detects differences in splicing at the intron level, which greatly improves performance when transcript reconstructions are inaccurate or incomplete. It identifies events directly from the alignment data, without relying on a reference gene annotation, and therefore can find and report novel events. MntJULiP can detect both differences in the introns’ splicing ratios (DSR), and changes in the abundance level of introns (DSA), and thus can capture alternative splicing variations in a comprehensive way. For DSA, it considers each intron individually and models read counts with a zero-inflated negative binomial (ZINB) distribution. For DSR, it groups introns sharing a splice site into a “bunch” and uses a Dirichlet multinomial (DM) distribution to simultaneously model all introns in a group (Supplementary Fig. S1). Additionally, MntJULiP has the ability to perform multiple comparisons simultaneously, which we showed was more accurate at capturing global differences in a time series or complex experiments [4]. We modified the Bayesian mixture models of MntJULiP to account for linear effects of covariates as described below.

### Covariate-augmented Bayesian models

We introduce covariate effects as linear components in the MntJULiP Bayesian models to account for extraneous attributes that could bias the analysis. Numerical covariates, such as age and weight, are centered and scaled, while categorical covariates, such as biological sex and ethnicity, are encoded into a numerical representation (0, 1, … K-1). Using the covariate-augmented models, we generate new adjusted counts and PSI values per sample, which can be used to generate global views of the alternative splicing variation as heatmaps or PCA plots using Jutils or other visualization tools.

#### The differential splicing abundance (DSA) model

This model tests an intron for differences in abundance among *K* conditions. Let *N* be the number of samples, *K* conditions, and *P* covariates (including the comparison). The covariate-augmented read count y of intron v in sample i follows a zero-inflated negative binomial distribution ZINB(*μ*_*k*_ + *x*_*i*_*β*_*k*_+ *a*_*k*_, θ) with mean *μ, K* coefficient column vectors *β*_*k*_ of length *P*, sample intercept *a*_*k*_, and *N* covariate row vectors *x*_*i*_ of length *P*. We re-fit the prior on the sample mean, previously introduced to capture the variances across different conditions and within individual samples, to a normal distribution: ${\mathrm{\mu }}$ ∼ N[${\mathrm{\hat{\mu}}}$, sqrt(${\mathrm{\hat{\mu }}}$)], and the prior on the dispersion parameter to an inverse Half Cauchy (HC) distribution: φ^−1^ ∼ sqrt[HC(0, 1)]. The zero-inflated enhanced negative binomial (ZINB) Bayesian model then is


\begin{eqnarray*}
Y = f\left( x \right) = \left\{ {\begin{array}{@{}*{1}{l}@{}} {0,\quad {\mathrm{with\ probability}}\ \pi ,}\\ {\mathrm{ NB}\left( {\mu + {{x}_i}{{\beta }_k} + {{a}_k}} \right),\quad \textrm{with}\ \textrm{probability}\ \left( {1 - \pi } \right).} \end{array}} \right.
\end{eqnarray*}

Maximum likelihood estimation is performed with respect to parameters *µ, β, a*, and *θ*, separately for the null and alternative models (i.e. samples generated from a single-condition and from a K-condition experiment, respectively) to obtain log likelihoods *L*(θ_0_) and *L*(θ_1_) for testing [4].

#### The differential splicing ratio (DSR) model

The model tests the competing introns within a “bunch” for differences in splicing ratios among conditions. The covariate-augmented read counts y_i1_, y_i2_, …, y_iM_ in sample i for a “bunch” with M introns follow a Dirichlet multinomial distribution with concentration parameters α_i1_, α_i2_, …, α_iM_, the M coefficient row vectors β_m_ of length P, the intercepts a_m_, N covariate column vectors x_i_ of length P, and “bunch” total n_iB_ = Σ_m′_y_im′_ [3]:


\begin{eqnarray*}
{{y}_{i1}}, {{y}_{i2}}, \ldots {{y}_{iM}}|{{n}_i}\sim \mathrm{ DM}\left( {{{n}_{iB,}}{{\alpha }_{i1}}{{p}_{i1}},\,{{\alpha }_{i2}}{{p}_{i2}}\, \ldots ,\,{{\alpha }_{iM}}{{p}_{iM}}} \right),
\end{eqnarray*}

\begin{eqnarray*}
{{p}_{im}} = exp\left( {{{x}_i}{{\beta }_m} + {{a}_m}} \right) / {{\Sigma }_{m\prime }}exp\left( {{{x}_i}{{\beta }_{m\text{'}}} + {{a}_{m\text{'}}}} \right).
\end{eqnarray*}

Maximum likelihood estimation is performed with respect to parameters *α, β*, and *a*, separately for the null and alternative models (excluding and including the condition column *x*, respectively) to obtain the log likelihoods *L*(θ_0_) and *L*(θ_1_) for testing [4].

#### Covariate-adjusted PSI and abundance estimates

Using the covariate-augmented models above, we generate new adjusted counts and Percent Spliced In (PSI) values per sample. After regressing out confounders, we obtain residuals *r*, applying a weakly informative prior *r* ∼ *N*(0, 10). For *abundance* estimates, *r* is estimated for the alternative model with the optimized *μ, β, a*, and *θ*:


\begin{eqnarray*}
y\sim\left\{ {\begin{array}{@{}*{1}{l}@{}} {0,\quad {\mathrm{with\ probability}}\ \pi, }\\ {NB\left( {\mu + {{x}_i}{{\beta }_k} + {{a}_k} + r,\theta } \right),\quad \textrm{with}\ \textrm{probability}\ \left( {1 - \pi } \right).} \end{array}} \right.
\end{eqnarray*}

For splicing ratios (PSI) estimates, *r* is estimated for the alternative model using the optimized *α, β*, and a [1]:


\begin{eqnarray*}
{{y}_{i1}}, {{y}_{i2}}, \ldots {{y}_{iM}}|{{n}_i}\sim \mathrm{ DM}\left( {{{n}_{iB,}}{{\alpha }_{i1}}{{p}_{i1}},\,{{\alpha }_{i2}}{{p}_{i2}}\, \ldots ,\,{{\alpha }_{iM}}{{p}_{iM}}} \right),
\end{eqnarray*}

\begin{eqnarray*}
{{p}_{im}} = exp({{x}_i}{{\beta }_m} + {{a}_m} + {{r}_i})/ {{\Sigma }_{k\prime }}exp\left( {{{x}_i}{{\beta }_{m\text{'}}} + {{a}_{m\text{'}}} + {{r}_i}} \right).
\end{eqnarray*}

The negative binomial and the Dirichlet multinomial models are implemented in PyStan, a Python package for Bayesian inference. Estimated counts were added to the output “intron_data.txt” file, and estimated PSI values were reported in a new file “group_data.txt,” complementing the existing raw count values.

### Differential splicing visualization with Jutils

Jutils [10] is a Python package for visualization of differential splicing events in the form of heatmaps, sashimi plots, and Venn diagrams of sets of differentially spliced genes. Jutils can be used with virtually any differential splicing detection tool and is specifically configured to work with the output of popular methods, including LeafCutter, MntJULiP, and rMATS. It converts the output of each tool into an intermediate TSV-formatted file to render the data in a unified format, described in [10]. This file is then sufficient to create visualizations, making it lightweight and very well suited for collaborations.

We extend Jutils and introduce new visualizations of PCA plots to identify potential unknown relationships among samples and to observe the effect of covariates on the data. PCA plots are generated from the splicing ratios (PSI) for DSR methods, or read counts for DSA methods, contained in the TSV file and imported from the output of differential splicing programs. As customizable features, input data can be filtered by significance, and data points can be differentiated by color, shape, and label based on conditions or covariates.

### Validation of covariate models on simulated data

To validate the covariate models, we simulated data for one “condition,” with values “control,” “disease,” and “stage2,” with one covariate, “biological sex,” with values “M” and “F” as described in [4]. Specifically, changes were simulated in the expression (DE) and/or the splicing ratio (DS) of genes. Changes in expression (DE) were simulated by either halving or doubling the expression level of the gene. Changes in splicing ratios (DS) were simulated by swapping the expression levels of the gene’s top two transcript isoforms.

For the *pairwise comparisons*, differences due to “condition” between the “control” and the “disease” states were simulated at 600 genes, including 200 DE, 200 DS, and 200 DE + DS genes. Differences in “biological sex” (covariate) were represented as changes in 300 genes, 100 from each of the DS, DE, and DE + DS categories. Consequently, the target gene set for the *DSR pairwise comparison* consists of the pooled 200 DS and 200 DS + DE genes differentially spliced between the “control” and “disease” states, while for the *DSA pairwise comparison* the target gene set is the set of 600 modified genes, 200 for each of the DS, DE, and DS + DE categories (see Supplementary Table S1).

For the *three-way comparisons*, changes at 100 of the previously modified genes were maintained, and additional changes between “disease” and “stage2” were made to a set of 200 additional genes not encountered previously, for each of the DE, DS, and DE + DS categories. Therefore, for the *DSR three-way comparison*, the target represents the 800 genes simulated as being DS or DE + DS between any of the “control,” “disease,” and “stage2” categories, while for the multi-way DSA comparison, the target is the full set of 1200 genes (400 DE, 400 DS, and 400 DE + DS) simulated to have changed between any of the “control,” “disease,” and “stage2” states (see Supplementary Table S2).

### Comparative program evaluation

Simulated reads were aligned to the human genome sequence GRCh38 using STAR v2.7.10a [12] and were analyzed for alternative splicing detection using the programs MntJULiP v.1.15.2, LeafCutter v.0.2.9 [3], DRIMSeq v.1.30.0 [8], and DEXSeq v.1.52.0 [9] for DSR, and with the differential gene expression tool DESeq2 v.1.42.1 [13] with *p*-val ≤ 0.05 and *q*-val ≤ 0.05 cutoffs for DSA, with and without accounting for covariates. For DRIMSeq, we used the “batch” variable to account for the “biological sex” covariate. MntJULiP, LeafCutter, DRIMSeq, and DESeq2 use “introns” as features in the comparisons, whereas DEXSeq is based on exons. Additionally, for DRIMSeq and DESeq2, we used as input the set of introns curated by MntJULiP’s intron extraction tool, “junc.” For each method, the set of genes with predicted events was used in the evaluation. Lastly, we use the term “accuracy” to refer to the overall correctness of the methods and employ conventional measures [sensitivity, Sn = TP/(TP + FN); precision, Pr = TP/(TP + FP), and the F-value, *F* = 2*Sn*Pr/(Sn + Pr)] to assess it.

### Analyses of GTEx data

Alignments of GTEx RNA-seq reads were those reported in [4], produced by HISAT2 [14] with RefSeq transcript annotations. Gene functional classification, and Gene Ontology (GO) and pathway enrichment analyses were performed with the tools DAVID [15] and Metascape [16], using a False Discovery Rate (FDR) < 0.1 for significance.

## Results

### Method evaluation on control data

To validate the covariate models, we simulated data for one “condition” variable, with values “control,” “disease,” and “stage2,” with one covariate, “biological sex,” with values “M” and ‘F.” For each condition, we generated 10 samples in each of the categories “condition” x “biological sex.” Starting from an empirical transcript expression matrix trained on an RNA-seq data set from lung fibroblasts (GenBank A# SRR493366) and using GENCODE v.41 as reference, we generated 11.5 million 100 bp long paired-end reads per sample from 2000 genes with two or more expressed isoforms. Changes in splicing were simulated as described in [4], separately for the DSR and the DSA models; detailed descriptions are provided in the “Materials and methods” section.

**Figure 1.**
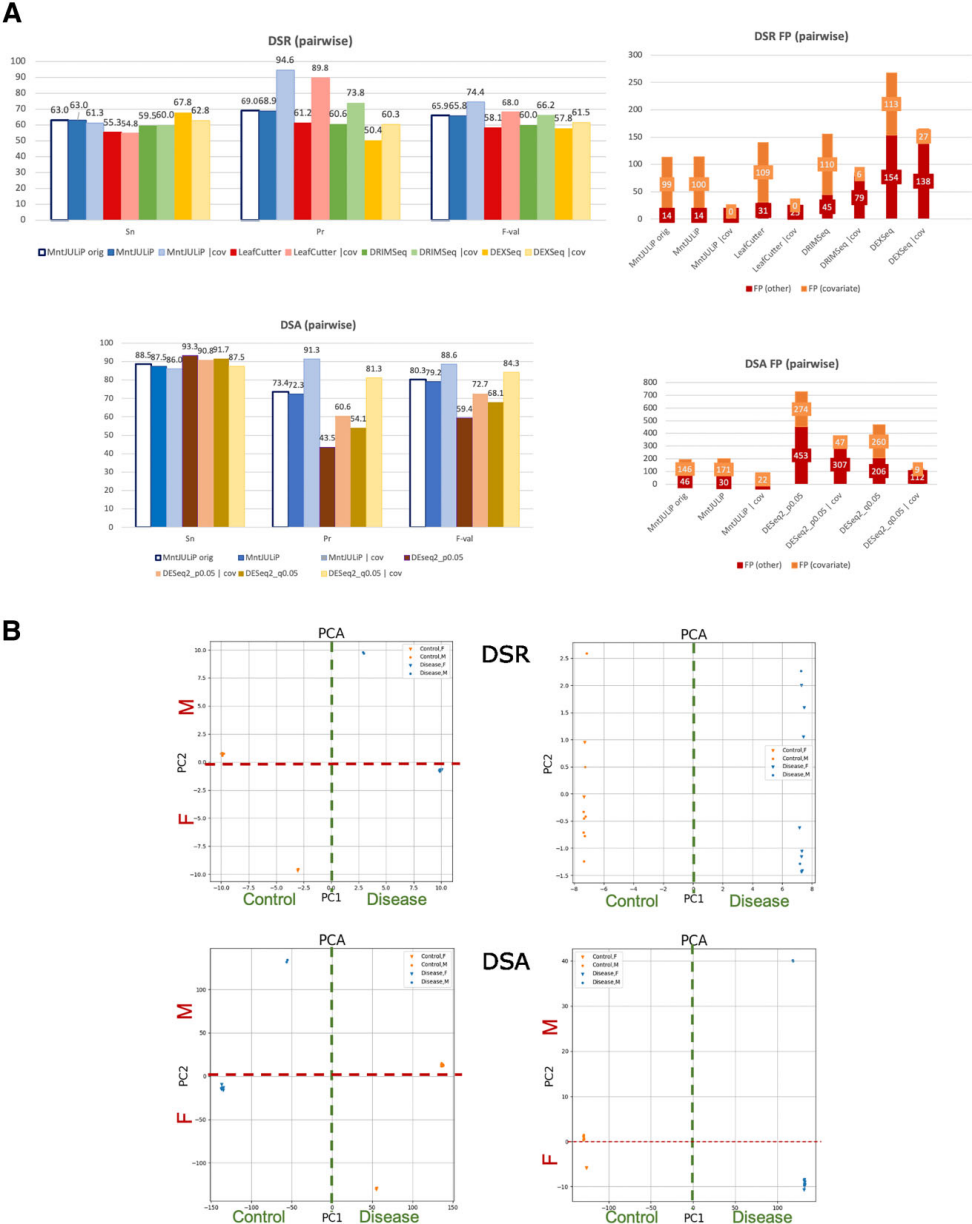
Evaluation of MntJULiP’s covariate function for pairwise comparison on simulated data. (**A**) MntJULiP was evaluated alongside LeafCutter, DRIMSeq, and DEXSeq for DSR comparisons (top), and DESeq2 (with *p*-val ≤ 0.05 and *q*-val ≤ 0.05 cutoffs) for DSA comparisons (bottom). On the left, performance was evaluated and shown using standard measures: Sn = TP/(TP + FN), Pr = TP/(TP + FP), and *F*-val = 2*Sn*Pr/(Sn + Pr). The breakdown of false positives (FPs) by covariate-related versus extrinsic factors is shown on the right. (**B**) PCA plots of samples generated with Jutils based on estimated PSI values, before and after covariate treatment. Top: DSR comparison; bottom: DSA comparison. Legend: circles, male (“M”) samples; inverted triangles, female (“F”) samples; orange, “control”; and blue, “disease.” Including “biological sex” as covariate in the models removes dependency along PC2 in the DSR comparison, while for the DSA comparison separation among “M” and “F” samples from the same category (“control,” “disease”) is drastically decreased.

We evaluated the performance of MntJULiP in splicing ratio (DSR) pairwise comparison on the simulated data, with and without covariates, alongside LeafCutter, DRIMSeq, and DEXSeq, which are *the only* other programs to implement covariates, in an imbalanced comparison of (8M, 2F) “control” versus (8F, 2M) “disease” samples (Fig. 1A). MntJULiP drastically reduced the false positives due to covariate bias while achieving sensitivity comparable to the original implementation, thus improving the accuracy. MntJULiP significantly outperforms all other programs as measured by the *F*-value, 0.744, followed by LeafCutter, 0.680, achieving the highest precision (0.945) and sensitivity comparable to the top program (0.613 versus DEXSeq’s 0.628). Further, the PCA plots of the estimated PSI values indicate that variation due to “biological sex” was correctly removed (Fig. 1B).

Similarly, MntJULiP outperforms DESeq2 in the DSA pairwise comparisons in overall accuracy, with an *F*-value of 0.886 compared to 0.843 and 0.727, respectively, for the two DESeq2 options. Once again, MntJULiP has the highest precision, 0.913, and near-best sensitivity, 0.860 versus 0.875 for DESeq2. While not entirely eliminating them, both programs significantly reduce the number of covariate-driven (and other) false positives (Fig. 1A and B). Similar results were obtained for the DSR and the DSA multiway comparisons (Supplementary Fig. S2), with MntJULiP consistently showing the highest precision and overall accuracy. (Note that LeafCutter does not allow for multi-way comparisons, whereas DRIMSeq allows for only one covariate, “batch.”) Therefore, MntJULiP models and removes biases due to covariates from the RNA-seq data to consistently achieve high accuracy, especially precision, and is more accurate than its competitors.

### Deconvoluting the effects of covariates on human frontal cortex splicing from GTEx RNA-seq data

To illustrate, we applied our methods to 1398 GTEx RNA-seq samples from 13 brain regions in three comparisons. The first comparison, among regions, revealed distinct groupings between the cerebellar, cortex, and basal ganglia regions, which did not change when accounting for the covariates “biological sex” and “age at death,” as was expected (Supplementary Fig. S3).

Second, we compared the 120 frontal cortex RNA-seq samples by age groups (“20s,” …, “70s”). Changes in the frontal cortex in aging contribute to sex-specific differences in the prevalence of neurological disorders [17]. More differences were observed with more distant age groups, consistent with reports of splicing deregulation with aging [18] (Supplementary Fig. S4). As an outlier, the higher number of events between the “20s” and “30s” groups may be due to the small number of samples in those categories (3 and 4, respectively). When “biological sex” was used as a covariate, a significant increase in differences was observed between the “20s” and “40s” groups, indicating a possible mark of sex-specific differentiation. As a mark of robustness, similar trends were observed when using different alignment tools, with and without using reference gene annotations in alignments; notably and expectedly, the use of annotation at the alignment stage increased the number of spliced alignments and, as a consequence, the number of predictions (Supplementary Fig. S5). Further investigating the “20s” versus “40s” comparison, accounting for the covariate increased the number of differentially spliced genes (931 versus 760 without covariate) and revealed additional disease, gene ontology, and pathway categories of enrichment (Supplementary Fig. S6). Specifically, while both comparisons identified “Chemdependency/Tobacco use disorder” as a significantly enriched disease class, “Neurogenesis” and “Exocytosis” as biological processes, “Kinase,” “Guanine nucleotide releasing factor,” “Transferase,” and “Actin binding” as molecular functions, and “Protein–protein interaction at synapses” and the brain-specific “Splicing factor NOVA regulated synaptic proteins” pathways, the covariate-aware comparison identified additional categories, including “Endocytosis,” “Insulin secretion,” “Signalling by RHO GTPases,” “VEGFA-VEGFR2 pathway,” as well as a class of 45 “RNA binding” genes, including several spliceosome and splicing regulatory proteins (CASC3, RP9, HNRNPDL, HRRNPUL1, PRPF3, PRPF38B, SRSF4, and SNRNPN) (Supplementary Fig. S7), suggesting differences in the splicing machinery between the two age groups. Further, it identified a group of seven proteins (DNM3, PRKCB, PLCB1, PRKACB, SLC8A1, AP2M1, and DNM1) in the enriched “Endocrine and other factor-regulated calcium reabsorption” KEGG pathway. Lastly, the fact that both comparisons point to “Chemdependency/Tobacco use disorder” as enriched category indicates the need to account for additional covariates, such as smoking status, to further reduce confounders.

**Figure 2.**
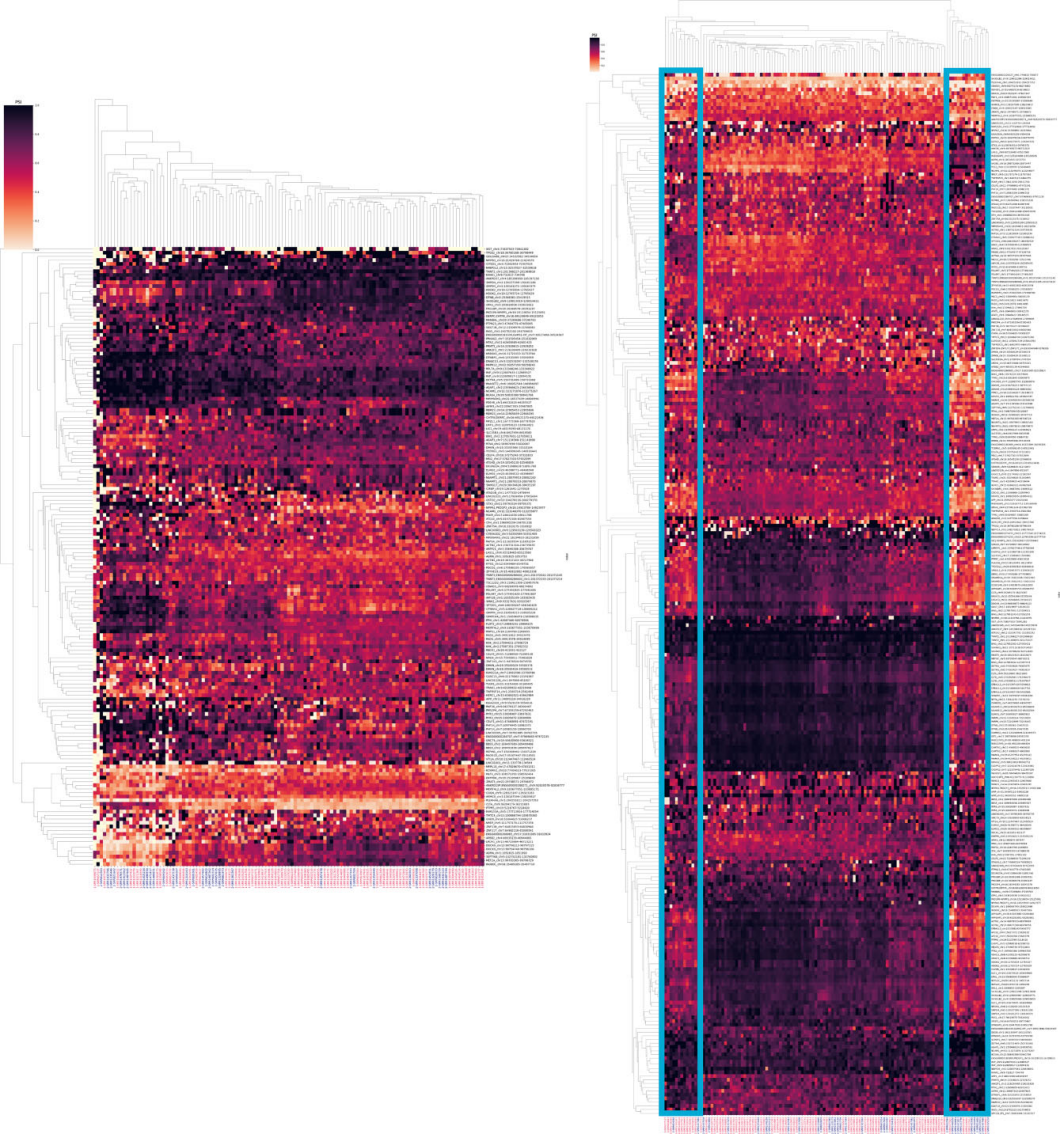
Differential splicing analyses of GTEx brain RNA-seq data with MntJULiP and Jutils. Jutils heatmaps of DSR events from the comparison between the frontal cortex female (“F”) and male (“M”) sample groups, without (left) and with (right) “age at death” as covariate. PSI intron values estimated with MntJULiP and uploaded into Jutils were plotted, with rows (events) and columns (samples) clustered using the “weighted” method and “cityblock” similarity metric. The covariate-adjusted heatmap shows a distinct supercluster marked with boxes.

Lastly, we compared the 83 male (“M”) and 37 female (“F”) frontal cortex samples to identify sex-specific differences in splicing. Jutils heatmaps of PSI values revealed a subgroup of 29 samples (13 “F” and 16 “M”) with a distinct alternative splicing pattern, which became evident when regressing for “age at death.” The 81 genes with distinguishing splicing patterns were enriched in categories including endocytosis, membrane trafficking, vesicle-mediated transport, adherens junctions, and brain-derived neurotrophic factor (BDNF) signaling, with broad roles in neuronal survival, differentiation, synaptic plasticity, cellular transport and communication, and tissue architecture. The subgroup over-represented females (13F:16M compared to 37F:83M for the entire set) and pointed to a probable distinct splicing program in a subset of individuals with aging (Fig. 2 and Supplementary Fig. S8). Similar DSA analyses of the frontal cortex data revealed distinguishing events, including at the non-coding X Inactive Specific Transcript (XIST) gene, which is involved in X chromosome inactivation in female early development processes, both without and with covariate modeling, thus supporting the ability of the program to identify and retain relevant genes (Supplementary Fig. S9). Therefore, as previously noted [4], DSR and DSA reflect different and complementary views and effects of alternative splicing on the transcriptional and functional outcomes.

To further validate and understand the effects of incorporating covariates, we functionally analyzed the sets of differentially spliced genes from the male-female comparisons above. We used Metascape, a platform for functional annotation of gene lists that combines over 40 independent knowledgebases, to comparatively analyze functional categories between the no-covariate comparison and when accounting for “age at death” as a confounding factor. For DSR, the age-covariate comparison increased the number of differential splicing events reported from 165 to 282, and the number of genes from 148 to 221 (Supplementary Fig. S10A), thus revealing classes of genes with splicing differences previously obscured by age imbalances between the cohorts. Metascape analysis identified gains in categories associated with sex differences in frontal cortex, including “Membrane organization,” “Brain-derived neurotrophic factor (BDNF) signaling,” “Regulation of synapses,” and “Endocytosis” (Supplementary Fig. S10B and references therein). Intriguingly, the only category to show a decrease in significance when age at death was used as covariate was regulation of messenger RNA (mRNA) splicing via the spliceosome, indicating that splicing differences at these genes could be more likely explained by differences in the “age” distribution between the male and female groups and, by extension, by the general aging process. This finding is consistent with previous reports implicating mRNA splicing, the spliceosome, and splicing regulatory factors in the aging process [18, 19]. It is further in alignment with our earlier finding of “RNA binding” as an enriched category in the “20s-versus-40s” age group comparisons when regressing out effects of biological sex (Supplementary Fig. S7).

Unlike for DSR above, the DSA covariate-adjusted comparison reduced the number of events identified from 90 to 32, and the number of genes from 69 to 22, indicating that the program likely removed genes whose differences in splicing could be accounted for by “age” differences between the male and female groups (Supplementary Fig. S11A). Validating our approach, the only category to show an increase in significance when regressing out donor age is “Extracellular matrix organization,” a potential factor of sex-based dimorphism in brain [20]. Conversely, several categories that were significant in both comparisons, including “Brain development” and “Cellular response to organic cyclic compound,” showed reduced significance after normalizing for “age.” Lastly, several categories, including “Interferon gamma signaling,” “Learning,” “Pathways of neurodegeneration,” and “Axon development,” appeared significant only in the no-covariate comparison. These clusters, related to inflammation, immune response, and neurodegeneration, particularly in Alzheimer’s disease, present sex-based differences that, however, occur specifically during the aging process, and therefore were correctly removed or reduced (Supplementary Fig. S11B).

## Discussion and conclusions

Confounders such as sex, age, and other biomedical attributes inherent to RNA-seq datasets from disease and population studies can bias bioinformatics analyses. Nevertheless, tools that can effectively account for their effects are lagging. The R-based tools DRIMSeq and DESeq2 implement sophisticated statistical models, but their accuracy depends on the quality of the input read count matrix, and running them requires specialized expertise. Additionally, DRIMSeq can only model one covariate (“batch”). Currently, LeafCutter is the only other specialized end-to-end method for RNA-seq data, from intron selection to differential splicing testing, that implements covariates; however, it is limited to DSR and to pairwise comparisons. MntJULiP and Jutils are user-friendly command-line tools for end-to-end differential splicing detection and visualization that implement robust intron selection, comprehensively model DSA and DSR differences while accounting for covariate effects, and enable multi-way comparisons. As with all other differential splicing detection methods, limitations include high variability with small numbers of samples and for events with low read counts. In our analyses, MntJULiP effectively removed covariate effects from both simulated and real data to uncover patterns of splicing variation, while being consistently more accurate than its competitors. In particular, analyses of GTEx RNA-seq data from the frontal cortex helped identify patterns of differential splicing across age groups and pointed to a distinct splicing program in a subgroup of individuals. To conclude, MntJULiP and Jutils are highly effective and efficient analysis tools for large-scale complex RNA-seq datasets with confounding factors, and can reveal new insights into disease and population.

## Supplementary Material

lqaf140_Supplemental_File

## Data Availability

The tools MntJULiP and Jutils are available from https://github.com/splicebox/MntJULiP and https://github.com/splicebox/Jutils, respectively. Archived versions of the software, scripts, alignment files of simulated data, and results from applying the tools to analysis data, including gene lists, are available from Zenodo (DOIs: 10.5281/zenodo.15875405 and 10.5281/zenodo.14984116).
