# Supplementary material for: MntJULiP and Jutils: differential splicing analysis of RNA-seq data with covariates
Source: NAR Genom Bioinform. 2025 Nov 3;7(4):lqaf140. doi: 10.1093/nargab/lqaf140 (PMC12582023; doi:10.1093/nargab/lqaf140)
Supplement: lqaf140_Supplemental_File [file lqaf140_supplemental_file.pdf]

SUPPLEMENTARY MATERIAL FOR THE ARTICLE:
"MNTJULIP AND JUTILS: DIFFERENTIAL SPLICING ANALYSIS OF RNA-SEQ DATA WITH COVARIATES"
BY W.W. LUI, G. YANG, Z. HE and L. FLOREA

Table of Contents:

Supplementary Tables

Table S1. Outline of the simulation model for DSA and DSR pairwise comparisons.
Table S2. Outline of the simulation model for DSA and DSR multi(k=3)-way comparisons.

Supplementary Figures

Figure S1. The MntJULiP intron-based feature models.
Figure S2. Evaluation of MntJULiP's DSR (A-C) and DSA (D-F) functions with covariates for multi(k=3)-way comparison.
Figure S3. Alternative splicing profiles of brain tissues, from the analysis of 1398 GTEx brain RNA-seq samples.
Figure S4. Distance matrices and 2D multidimensional scaling maps of age group DSR comparisons in GTEx frontal cortex data, without and with 'biological sex' as covariate.
Figure S5. Robustness of program with different aligners, and with or without a reference gene annotation.
Figure S6. Gene functional analysis and pathway enrichment of DSR genes from the comparison between the '20s' and '40s' age groups, with and without using 'biological sex' as covariate.
Figure S7. List of RNA binding proteins from the GO molecular function enrichment analysis, unique to the 'sex-cov' '20s-vs-40s' comparison.
Figure S8. Jutils PCA plots (PC 1-5) for the male-vs-female differential splicing (DSR) analysis.
Figure S9. Jutils heatmaps of DSA events differentially spliced between 'F' and 'M' categories in GTEx frontal cortex data.
Supplementary Figure S10. Comparative gene functional analysis of DSR genes in the 'biological sex' comparison of GTEx frontal cortex samples.
Supplementary Figure S11. Comparative gene functional analysis of DSA genes in the 'biological sex' comparison of GTEx frontal cortex samples.

Supplementary Table S1. Outline of simulation model for DSA and DSR pairwise comparisons.

Condition\Covariate*	M	F
Control	0	100x(DS)+100x(DE)+100x(DS+DS)
Disease	200d(DS)+200d(DE)+200d(DE+DS)	100x(DS)+100x(DE)+100x(DE+DS)<br>200d(DS)+200d(DS)+200d (DE+DS)

*Genes modified versus reference (M,Control): 600 genes differentiate based on disease status ('d'), and 300 non-overlapping genes based on biological sex ('x').

Supplementary Table S2. Outline of simulation model for DSA and DSR multi(k=3)-way comparisons. Genes marked as (DS) and (DE+DS) are used to evaluate the DSR function, and all categories (DS), (DE) and (DE+DS) are used in assessing the DSA function.

Condition\Covariate *	M	F
Control	0	100x(DS)+100x(DE)+100x(DE+DS)
Disease	100d(DS)+100d(DE)+100d(DE+DS)	100x(DS)+100x(DE)+100x(DE+DS)<br>100d(DS)+100d(DE)+100d(DE+DS)
Stage2 & Disease	100ds2(DS)+100ds2(DE)+100ds2(DE+DS)	100x(DS)+100x(DE)+100x(DE+DS)<br>100ds2(DS)+100ds2(DE)+100ds2(DE+DS)
Stage2	200s2(DS)+200s2(DE)+200s2(DE+DS)	100x(DS)+100x(DE)+100x(DE+DS)<br>200s2(DS)+200s2(DE)+200s2(DE+DS)

*Genes modified versus reference (M,Control): 1,200 genes differentiate based on disease status ('d'- perturbed in 'disease' state only, 'ds2' – perturbed in both the 'disease' and 'stage2' states, and 's2' – perturbed in 'stage2' only); and 300 non-overlapping genes based on biological sex ('x'). Parts of the table highlighted in gray represent genes used in the pairwise comparisons (**Supplementary Table S1**).

Supplementary Figure S1. The MntJULiP intron-based feature models. *Left*, differential splicing abundance (DSA): each intron is analyzed individually, and the expression (abundance) level is compared between conditions. *Right*, differential splicing ratio (DSR): introns that share a splice junction ('bunch') are collectively analyzed, and the PSI values for the introns are compared between conditions. Shown are: an individual exon in a three-condition (C1, C2, C3) experiment, in the DSA diagram, and a three-intron 'bunch' in a two-condition (C1, C2) experiment, in the DSR diagram.

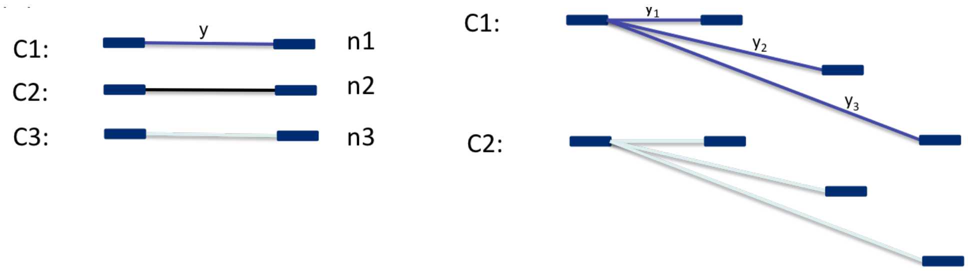

$PSI(y_i) = y_i / (y_1+y_2+y_3)$

(Percent Splicing Inclusion)

Supplementary Figure S2. Evaluation of MntJULiP's DSR (A-C) and DSA (D-F) functions with covariates for multi(k=3)-way comparison, and by comparison to similar tools: LeafCutter, DRIMSeq and DEXSeq for the DSR function, and DESeq2 for DSA operation. (A,D) Performance evaluation: Sn=TP/(TP+FN), Pr=TP/(TP+FP), F-val=2*Sn*Pr/(Sn+Pr). (B,E) Breakdown of FPs by covariate versus extrinsic factors. (C,F) PCA plots of samples based on PSI values (C) and intron abundance (F), respectively, generated with MntJULiP before (left) and after (right) adjustment for covariate. Before covariate adjustment: samples cluster by 'condition' x 'biological sex'. After covariate adjustment: samples within each 'condition' category no longer separated by 'biological sex'. (N.b., LeafCutter cannot be used in multi-way comparisons and was excluded.)

(A)

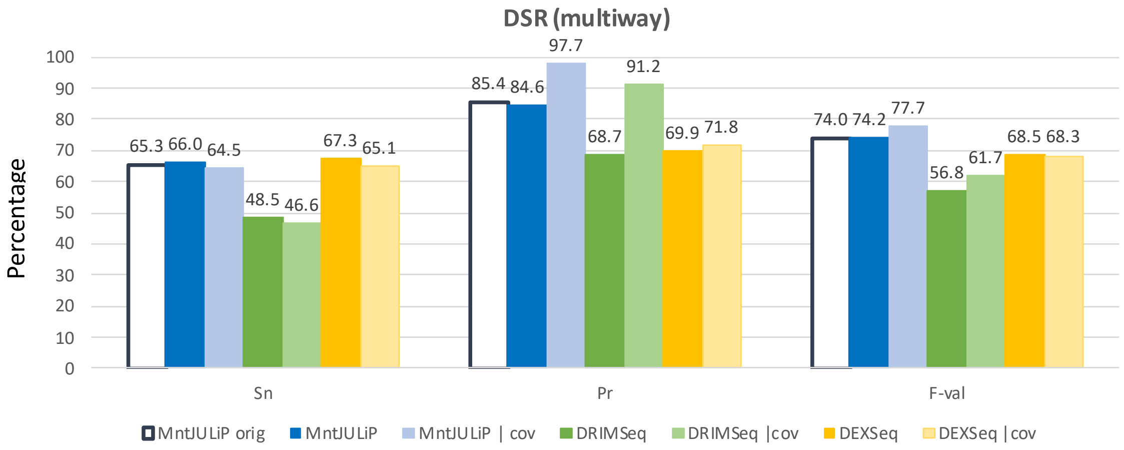

(B)

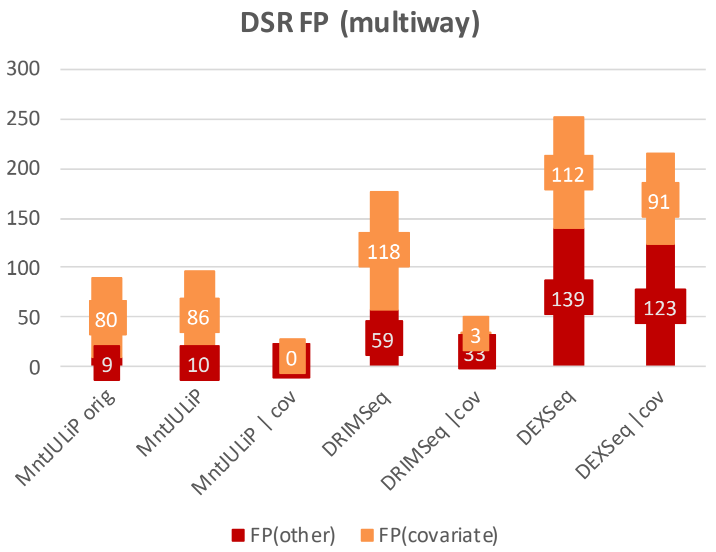

(C)

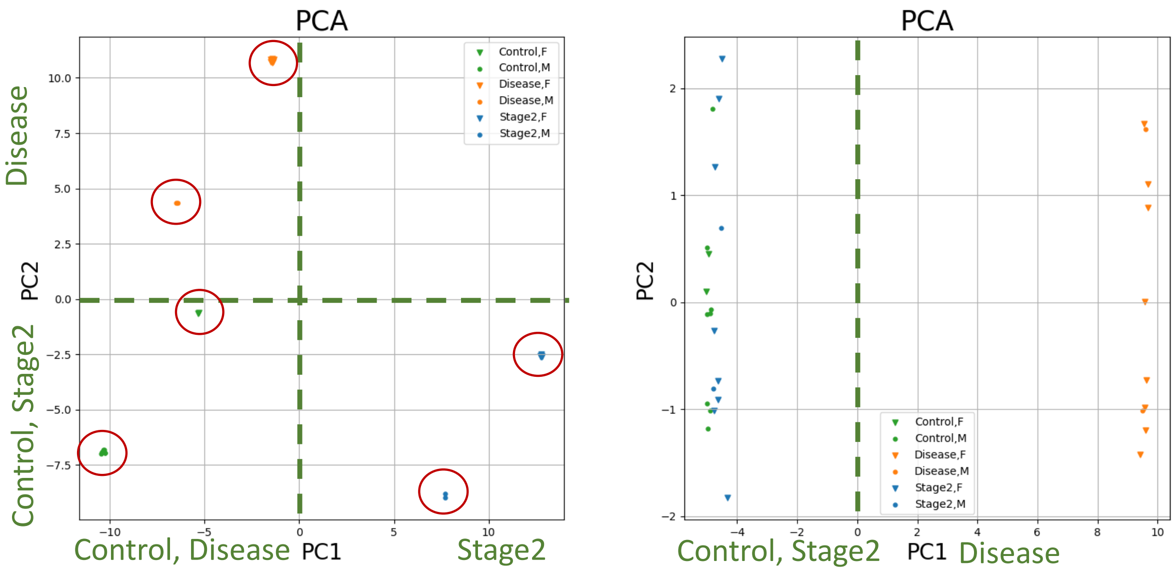
PCA
Disease
Control, Stage2
PC2
PC1
Control, Disease
Stage2
Control,F
Control,M
Disease,F
Disease,M
Stage2,F
Stage2,M
PCA
PC2
PC1
Control, Stage2
Disease

(D)

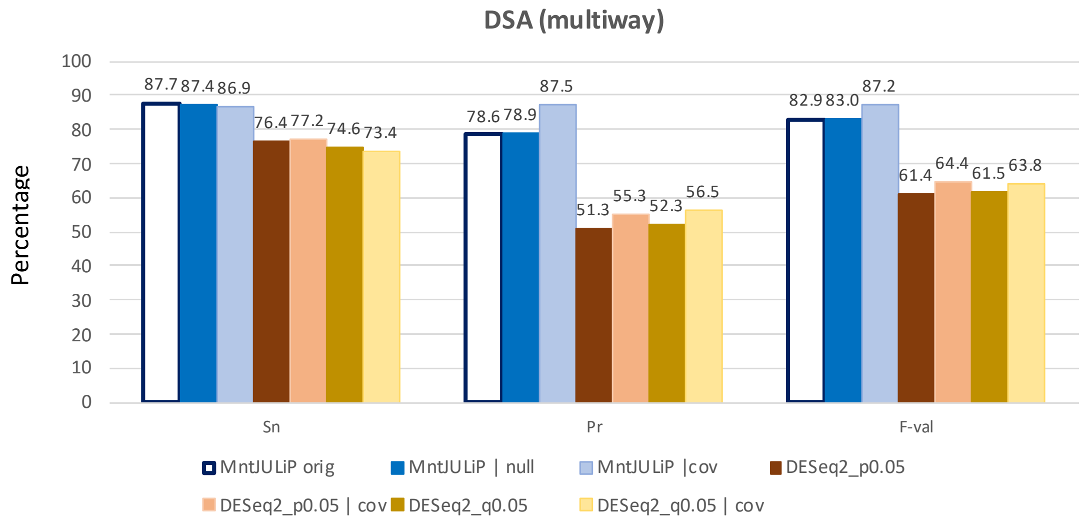
DSA (multiway)
Percentage
Sn
Pr
F-val
87.7
87.4
86.9
76.4
77.2
74.6
73.4
78.6
78.9
87.5
51.3
55.3
52.3
56.5
82.9
83.0
87.2
61.4
64.4
61.5
63.8
MntJULiP orig
MntJULiP | null
MntJULiP |cov
DESeq2_p0.05
DESeq2_p0.05 | cov
DESeq2_q0.05
DESeq2_q0.05 | cov

(E)

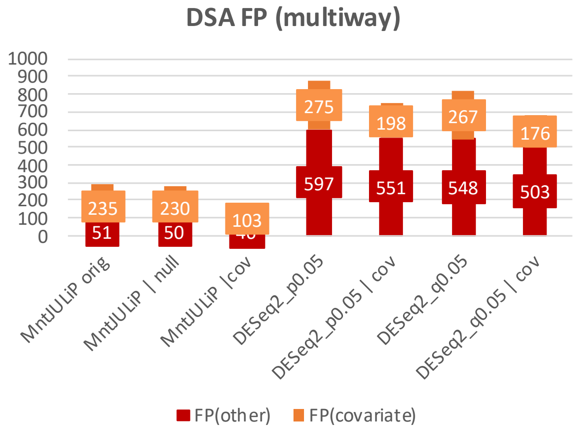
DSA FP (multiway)
235
51
230
50
103
275
597
198
551
267
548
176
503
MntJULiP orig
MntJULiP | null
MntJULiP |cov
DESeq2_p0.05
DESeq2_p0.05 | cov
DESeq2_q0.05
DESeq2_q0.05 | cov
FP(other)
FP(covariate)

(F)

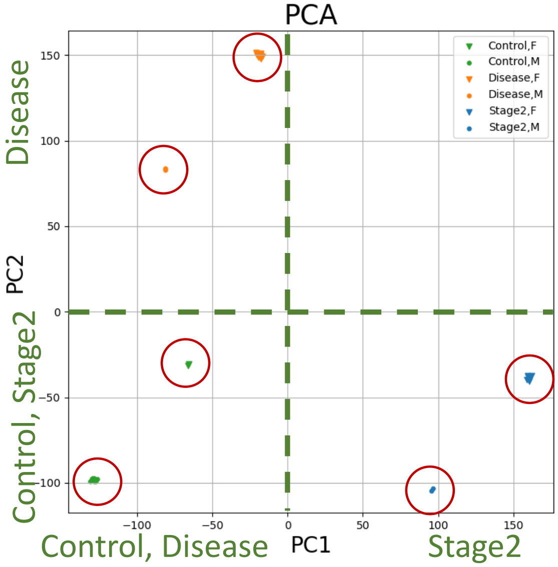
PCA
Disease
PC2
Control, Stage2
Control, Disease
PC1
Stage2
Control,F
Control,M
Disease,F
Disease,M
Stage2,F
Stage2,M
150
100
50
0
−50
−100
−100
−50
0
50
100
150

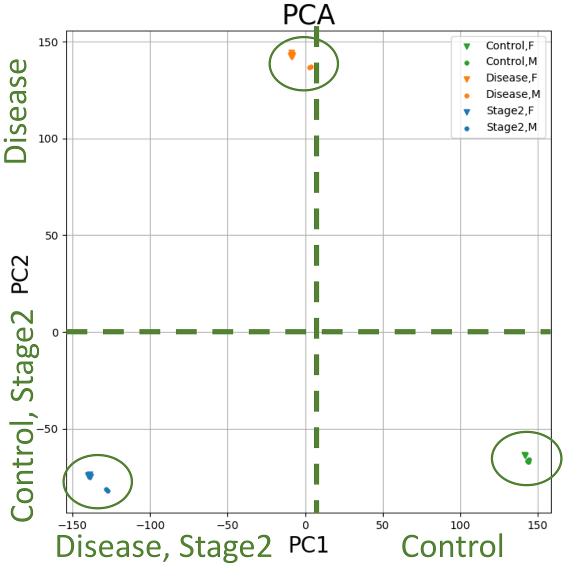
PCA
Disease
PC2
Control, Stage2
Disease, Stage2
PC1
Control
Control,F
Control,M
Disease,F
Disease,M
Stage2,F
Stage2,M
150
100
50
0
−50
−150
−100
−50
0
50
100
150

Supplementary Figure S3. Alternative splicing profiles of brain tissues, from differential splicing analysis of 1398 GTEx brain RNA-seq samples. (A) Distance matrices and 3D multidimensional scaling (MDS, or Principal Coordinate Analysis, PCoA) maps constructed from DSR differential splicing events: (top) without covariate treatment, (middle) with 'biological sex' as covariate, and (bottom) with 'age at death' as covariate. (B) Similarly, for DSA. Abbreviations: *AMG* – Amygdala, *ACX* – Anterior cingulate cortex BA24, *CRB* – Cerebellum, *CRH* – Cerebellar hemisphere, *CX* – Cortex, *CBG* – Caudate basal ganglia, *FCX* – Frontal cortex, *HIP* – Hippocampus, *HYP* – Hypothalamus, *PBG* – Putamen basal ganglia, *NBG* – Nucleus accumbens basal ganglia, *SNG* – Substantia nigra, *SPC* – Spinal cord cervical c1. Events with p-val<0.05 and |dPSI|>=0.2 (DSR), and p-val<0.05 and |l2FC|>=2.0 (DSA) were selected for these representations.

(A)

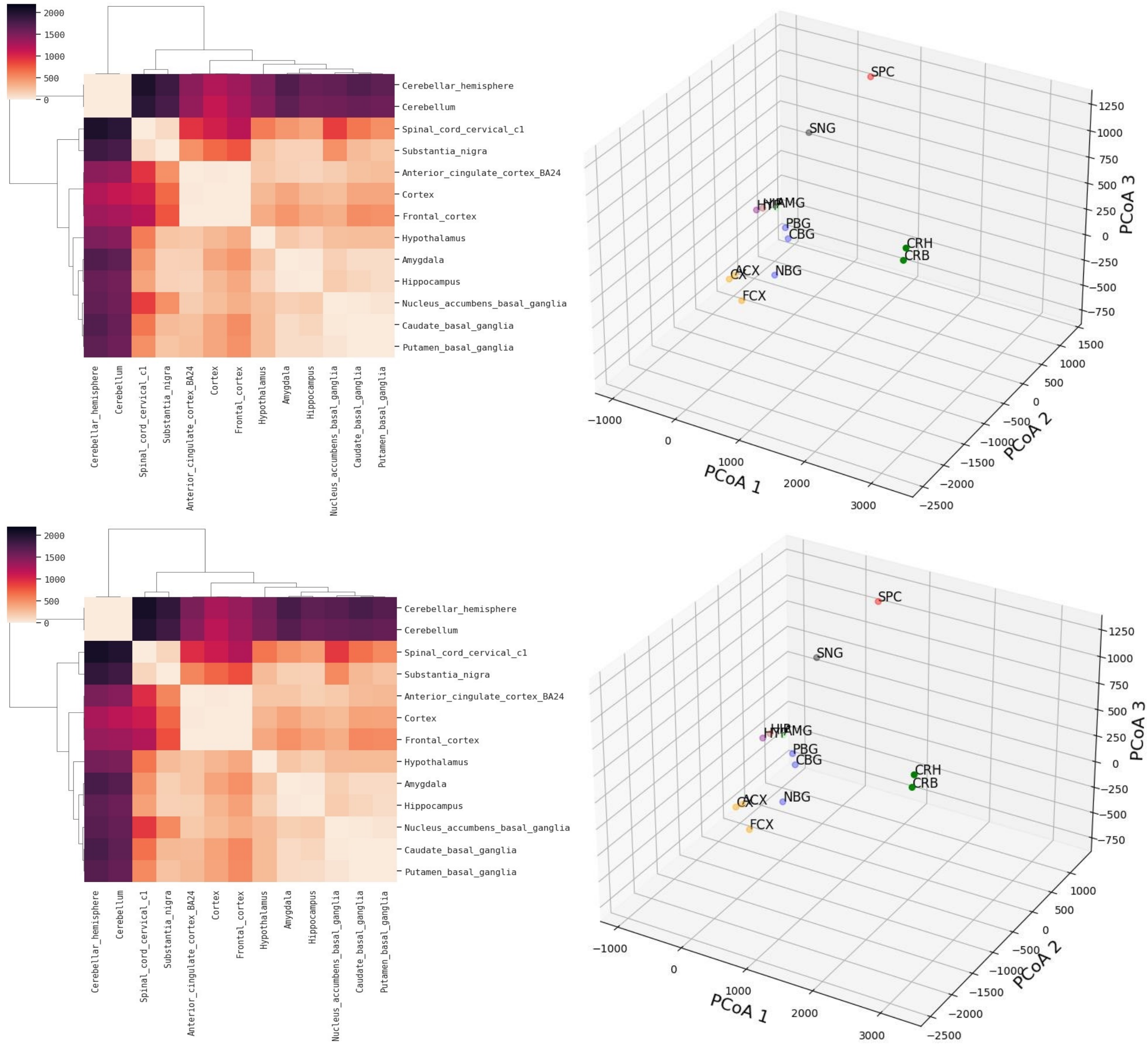

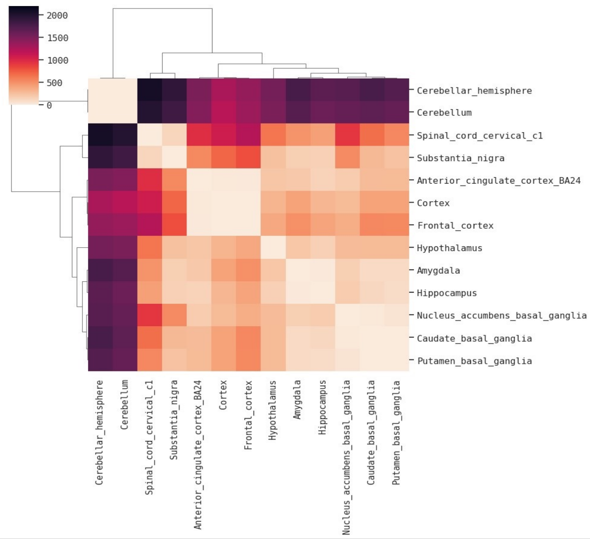

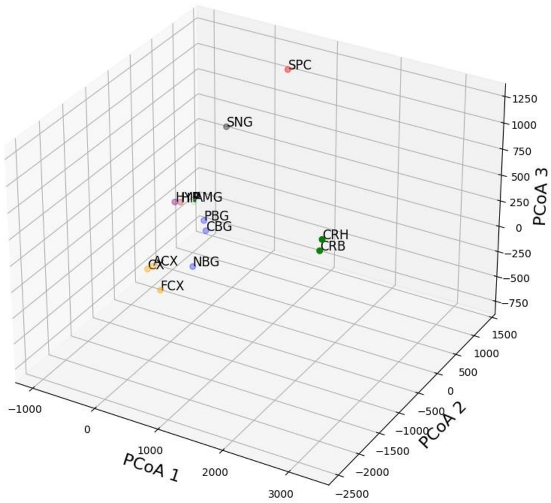

Tissue/region comparison (DSR) - distance matrix, no covariate													
	AMG	ACX	CBG	CRH	CRB	CX	FCX	HIP	HYP	NBG	PBG	SPC	SNG
AMG		201	94	1766	1690	414	494	17	213	164	95	475	162
ACX	201		265	1493	1446	18	9	151	213	183	279	959	537
CBG	94	265		1767	1648	413	536	122	273	25	3	651	281
CRH	1766	1493	1767		6	1265	1371	1669	1535	1687	1688	2053	1905
CRB	1690	1446	1648	6		1181	1329	1588	1490	1605	1608	1980	1825
CX	414	18	413	1265	1181		3	320	314	279	409	1088	712
FCX	494	9	536	1371	1329	3		405	374	340	529	1221	808
HIP	17	151	122	1669	1588	320	405		160	188	94	424	154
HYP	213	213	273	1535	1490	314	374	160		266	271	618	255
NBG	164	183	25	1687	1605	279	340	188	266		48	911	532
PBG	95	279	3	1688	1608	409	529	94	271	48		538	235
SPC	475	959	651	2053	1980	1088	1221	424	618	911	538		117
SNG	162	537	281	1905	1825	712	808	154	255	532	235	117	

Tissue/region comparison (DSR) - distance matrix, 'sex' covariate													
	AMG	ACX	CBG	CRH	CRB	CX	FCX	HIP	HYP	NBG	PBG	SPC	SNG
AMG		202	94	1745	1672	401	495	14	210	161	90	483	166
ACX	202		265	1491	1431	17	9	143	209	181	278	953	528
CBG	94	265		1747	1637	407	533	118	273	24	4	645	281
CRH	1745	1491	1747		7	1250	1369	1658	1527	1668	1695	2058	1900
CRB	1672	1431	1637	7		1182	1326	1579	1484	1595	1604	1979	1808
CX	401	17	407	1250	1182		3	304	305	274	406	1080	703
FCX	495	9	533	1369	1326	3		395	377	346	527	1210	801
HIP	14	143	118	1658	1579	304	395		149	177	92	424	155
HYP	210	209	273	1527	1484	305	377	149		265	272	621	246
NBG	161	181	24	1668	1595	274	346	177	265		46	914	519
PBG	90	278	4	1695	1604	406	527	92	272	46		538	235
SPC	483	953	645	2058	1979	1080	1210	424	621	914	538		117
SNG	166	528	281	1900	1808	703	801	155	246	519	235	117	

Tissue/region comparison (DSR) - distance matrix, 'age' covariate													
	AMG	ACX	CBG	CRH	CRB	CX	FCX	HIP	HYP	NBG	PBG	SPC	SNG
AMG		220	96	1747	1670	393	496	16	214	163	89	481	167
ACX	220		265	1489	1438	17	8	163	213	184	284	960	547
CBG	96	265		1762	1638	405	532	117	273	23	3	648	280
CRH	1747	1489	1762		6	1252	1355	1653	1524	1680	1696	2041	1902
CRB	1670	1438	1638	6		1169	1316	1581	1481	1599	1594	1959	1818
CX	393	17	405	1252	1169		3	310	302	278	400	1076	707
FCX	496	8	532	1355	1316	3		415	370	347	528	1221	809
HIP	16	163	117	1653	1581	310	415		161	183	92	409	154
HYP	214	213	273	1524	1481	302	370	161		268	272	625	264
NBG	163	184	23	1680	1599	278	347	183	268		49	910	530
PBG	89	284	3	1696	1594	400	528	92	272	49		535	235
SPC	481	960	648	2041	1959	1076	1221	409	625	910	535		119
SNG	167	547	280	1902	1818	707	809	154	264	530	235	119	

(B)

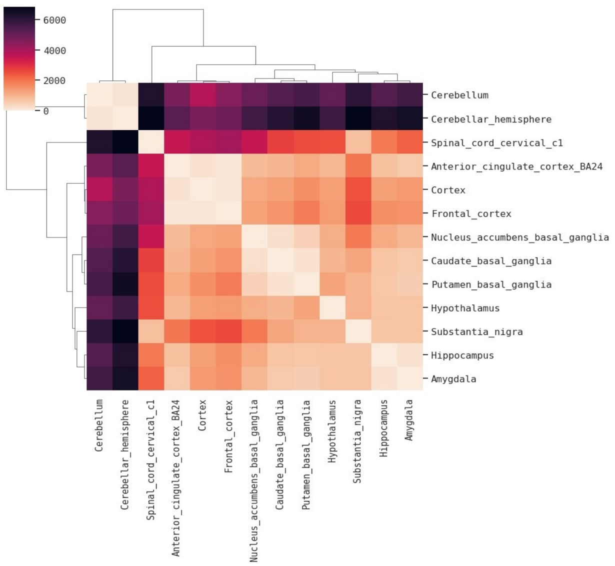

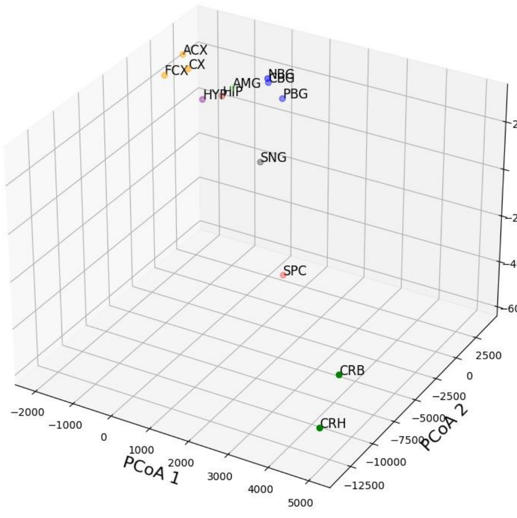

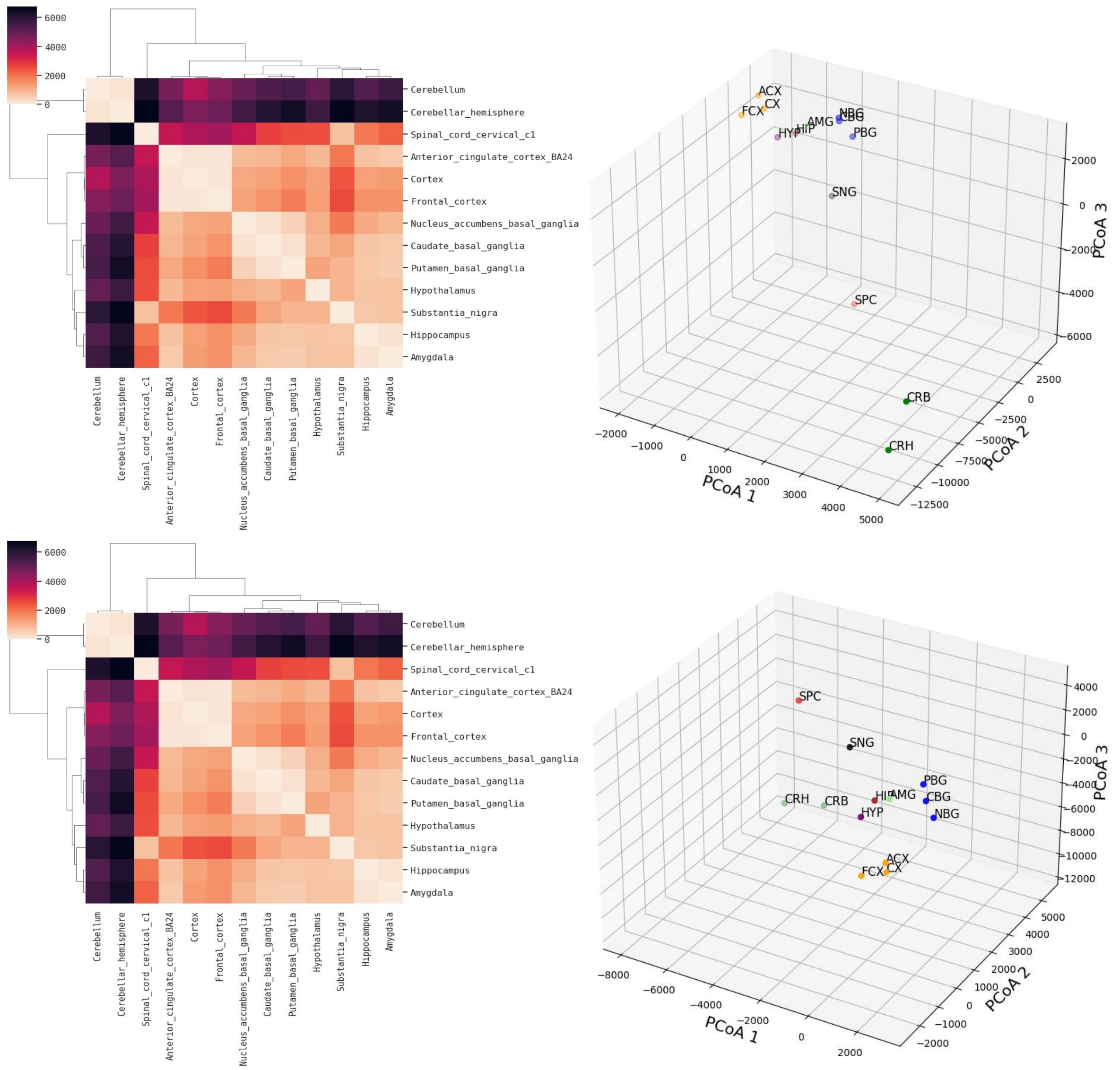

Tissue/region comparison (DSA) - distance matrix, no covariate

	AMG	ACX	CBG	CRH	CRB	CX	FCX	HIP	HYP	NBG	PBG	SPC	SNG
**AMG**		612	601	6500	5689	1417	1533	164	718	924	550	2254	703
**ACX**	612		956	5282	4738	166	101	765	924	876	1150	3528	1932
**CBG**	601	956		6177	5398	1303	1507	700	953	194	176	2722	1214
**CRH**	6500	5282	6177		158	4699	4901	6281	5731	5694	6534	6850	6829
**CRB**	5689	4738	5398	158		3786	4511	5376	5096	4991	5540	6299	6073
**CX**	1417	166	1303	4699	3786		91	1334	1312	1162	1594	3893	2472
**FCX**	1533	101	1507	4901	4511	91		1588	1367	1273	1873	4083	2605
**HIP**	164	765	700	6281	5376	1334	1588		711	1149	657	1905	678
**HYP**	718	924	953	5731	5096	1312	1367	711		1076	1254	2520	1007
**NBG**	924	876	194	5694	4991	1162	1273	1149	1076		471	3513	1913
**PBG**	550	1150	176	6534	5540	1594	1873	657	1254	471		2556	991
**SPC**	2254	3528	2722	6850	6299	3893	4083	1905	2520	3513	2556		782
**SNG**	703	1932	1214	6829	6073	2472	2605	678	1007	1913	991	782	

Tissue/region comparison (DSA) - distance matrix, 'sex' covariate

	AMG	ACX	CBG	CRH	CRB	CX	FCX	HIP	HYP	NBG	PBG	SPC	SNG
**AMG**		600	583	6427	5643	1381	1516	157	707	920	544	2235	695
**ACX**	600		936	5230	4720	157	92	732	902	865	1132	3509	1930
**CBG**	583	936		6084	5357	1260	1494	677	919	174	167	2693	1203
**CRH**	6427	5230	6084		150	4671	4848	6206	5682	5614	6464	6766	6736
**CRB**	5643	4720	5357	150		3770	4478	5317	5050	4945	5511	6258	6034
**CX**	1381	157	1260	4671	3770		83	1266	1281	1145	1545	3852	2438
**FCX**	1516	92	1494	4848	4478	83		1528	1355	1256	1840	4032	2575
**HIP**	157	732	677	6206	5317	1266	1528		694	1103	638	1907	650
**HYP**	707	902	919	5682	5050	1281	1355	694		1053	1234	2498	981
**NBG**	920	865	174	5614	4945	1145	1256	1103	1053		453	3486	1897
**PBG**	544	1132	167	6464	5511	1545	1840	638	1234	453		2540	967
**SPC**	2235	3509	2693	6766	6258	3852	4032	1907	2498	3486	2540		762
**SNG**	695	1930	1203	6736	6034	2438	2575	650	981	1897	967	762	

Tissue/region comparison (DSA) - distance matrix, 'age' covariate

	AMG	ACX	CBG	CRH	CRB	CX	FCX	HIP	HYP	NBG	PBG	SPC	SNG
**AMG**		596	583	6443	5666	1374	1515	165	700	912	536	2223	688
**ACX**	596		935	5244	4726	156	96	735	897	870	1141	3501	1918
**CBG**	583	935		6089	5366	1258	1483	679	924	174	168	2698	1199
**CRH**	6443	5244	6089		153	4668	4845	6211	5680	5611	6444	6765	6714
**CRB**	5666	4726	5366	153		3767	4484	5331	5043	4951	5511	6244	6013
**CX**	1374	156	1258	4668	3767		84	1280	1276	1138	1549	3862	2412
**FCX**	1515	96	1483	4845	4484	84		1544	1335	1247	1842	4040	2569
**HIP**	165	735	679	6211	5331	1280	1544		698	1114	639	1899	647
**HYP**	700	897	924	5680	5043	1276	1335	698		1057	1239	2489	976
**NBG**	912	870	174	5611	4951	1138	1247	1114	1057		465	3488	1879
**PBG**	536	1141	168	6444	5511	1549	1842	639	1239	465		2529	965
**SPC**	2223	3501	2698	6765	6244	3862	4040	1899	2489	3488	2529		759
**SNG**	688	1918	1199	6714	6013	2412	2569	647	976	1879	965	759	

Supplementary Figure S4. Distance matrices (A, B) and 2D multidimensional scaling (MDS, or Principal Coordinate Analysis, PCoA) maps of age group DSR comparisons (C) in GTEx frontal cortex data, without (left) and with (right) 'biological sex' as covariate, using p-val<0.05 and |dPSI|>0.2 for event selection. Using 'biological sex' as covariate, PcoA1 explains more of the variability in the data (79% compared to 75.5%). Age groups ('20s', '30s', ..., '70s') are shown along the sides of the matrix.

(A)

Age group comparison - distance matrix, no covariate

	**20s**	**30s**	**40s**	**50s**	**60s**	**70s**
**20s**		545	206	193	479	672
**30s**	545		154	159	159	425
**40s**	206	154		22	82	167
**50s**	193	159	22		6	90
**60s**	479	159	82	6		71
**70s**	672	425	167	90	71	

Age group comparison - distance matrix, 'sex'-covaria

	**20s**	**30s**	**40s**	**50s**	**60s**	**70s**
**20s**		550	355	167	444	672
**30s**	550		179	142	142	375
**40s**	355	179		21	79	157
**50s**	167	142	21		4	124
**60s**	444	142	79	4		56
**70s**	672	375	157	124	56	

(B)

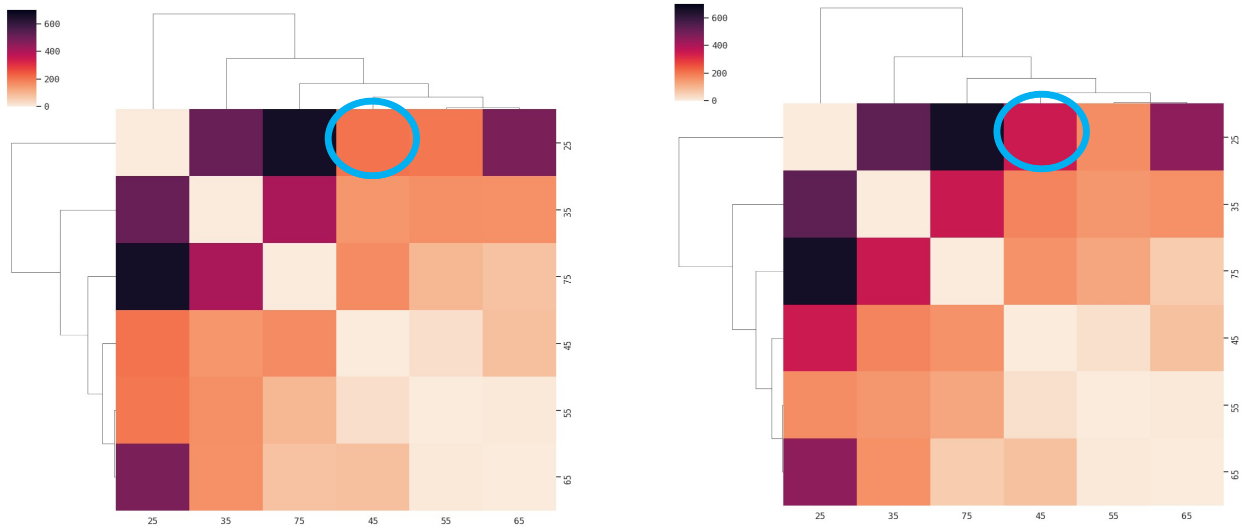

(C)

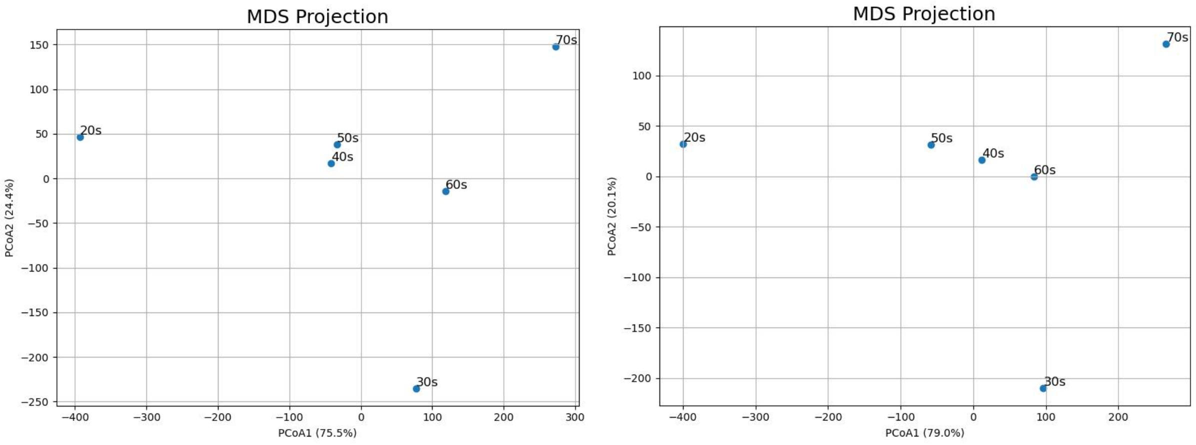

Figure S5. Robustness of program with different aligners, and with or without reference gene annotations, illustrated on the age group comparisons. (A) Distance matrices of MntJULiP DSR genes, starting from alignments generated with STAR, without (left) and with (right) reference gene annotations (CHESS+GENCODE) at the alignment step. The matrices reflect similar patterns of increasing differences with more distant age groups and a significant increase in the number of DSR genes for the '20s-vs-40s' comparison when using 'sex' as covariate, as observed previously for HISAT2 with RefSeq gene annotations (**Supplementary Figure S4 A**). (B) Bar plot of the numbers of differentially spliced genes for all comparisons, with the three alignment strategies. Leveraging transcriptome information slightly increases the number of identified events and DSR genes, by increasing the intron read support levels. Also, HISAT2 further increases the ability to identify DSR genes; however, the more significant increases are observed for comparisons with low numbers of samples ('20s-vs-30s', '20s-vs-70s' and '30s-vs-70s'; 3 or 4 samples each) and are likely to have resulted from variability in sampling. (C) Consistency between the gene sets predicted with different alignment strategies, measured by the number and percentage of common genes.

(A)

**STAR, no annotation**						
**DSR, Mode: no-covariate**	**20s**	**30s**	**40s**	**50s**	**60s**	**70s**
20s		288	164	146	377	426
30s	288		111	98	110	204
40s	164	111		3	53	116
50s	146	98	3		0	64
60s	377	110	53	0		34
70s	426	204	116	64	34	
**DSR, Mode: sex-covariate**	**20s**	**30s**	**40s**	**50s**	**60s**	**70s**
20s		282	240	128	350	426
30s	282		139	92	115	186
40s	240	139		6	51	86
50s	128	92	6		0	93
60s	350	115	51	0		36
70s	426	186	86	93	36	

**STAR, CHESS+GENCODE**						
**DSR, Mode: no-covariate**	**20s**	**30s**	**40s**	**50s**	**60s**	**70s**
20s		308	170	159	445	464
30s	308		133	116	123	229
40s	170	133		7	68	136
50s	159	116	7		2	72
60s	445	123	68	2		45
70s	464	229	136	72	45	
**DSR, Mode: sex-covariate**	**20s**	**30s**	**40s**	**50s**	**60s**	**70s**
20s		326	276	122	404	464
30s	326		145	113	119	204
40s	276	145		8	64	103
50s	122	113	8		1	107
60s	404	119	64	1		43
70s	464	204	103	107	43	

(B)

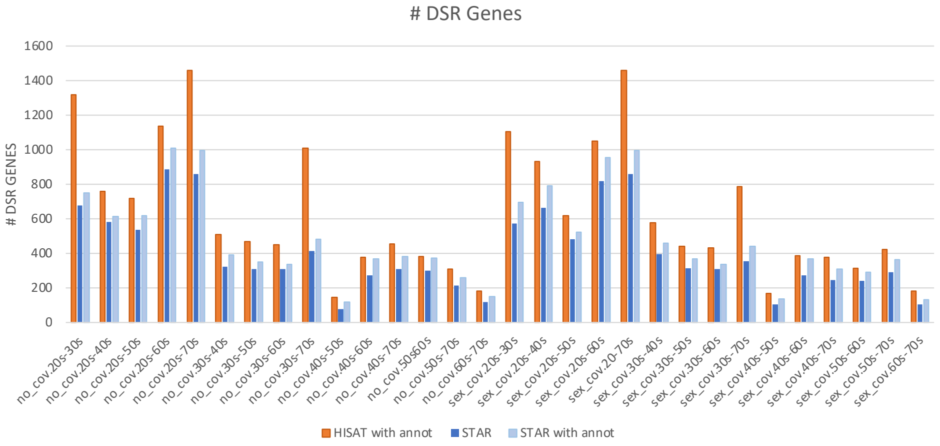

(C)

No-covariate

DSR Genes shared between STAR vs STAR+annot

Pct\#Genes	20s	30s	40s	50s	60s	70s
20s	.	388	350	366	670	617
30s	57%	.	160	170	178	219
40s	60%	49%	.	37	197	186
50s	68%	55%	47%	.	211	121
60s	75%	57%	72%	70%	.	67
70s	72%	53%	60%	56%	57%	.

HISAT+annot vs STAR

Pct/#Genes	**20s**	**30s**	**40s**	**50s**	**60s**	**70s**
20s	.	324	302	307	598	539
30s	48%	.	121	138	139	181
40s	52%	37%	.	33	178	151
50s	57%	45%	42%	.	185	100
60s	67%	45%	65%	61%	.	47
70s	63%	44%	49%	47%	40%	.

HISAT+annot vs STAR+annot

Pct/#Genes	20s	30s	40s	50s	60s	70s
20s	.	446	359	391	748	666
30s	59%	.	206	206	199	278
40s	59%	53%	.	66	254	212
50s	63%	59%	57%	.	269	159
60s	74%	59%	69%	72%	.	84
70s	67%	58%	55%	62%	55%	.

With 'biological sex' as covariate

STAR vs STAR+annot

Pct\#Genes	20s	30s	40s	50s	60s	70s
20s	.	363	446	315	624	617
30s	63%	.	204	172	174	213
40s	67%	52%	.	52	191	127
50s	66%	55%	50%	.	162	178
60s	76%	56%	70%	67%	.	58
70s	72%	60%	52%	61%	55%	.

HISAT+annot vs STAR

Pct/#Genes	20s	30s	40s	50s	60s	70s
20s	.	290	375	266	552	539
30s	51%	.	162	139	142	165
40s	56%	41%	.	45	177	106
50s	55%	44%	43%	.	143	151
60s	68%	46%	65%	59%	.	51
70s	63%	46%	43%	52%	49%	.

HISAT+annot vs STAR+annot

Pct/#Genes	20s	30s	40s	50s	60s	70s
20s	.	392	495	315	681	666
30s	56%	.	250	203	190	258
40s	63%	55%	.	75	258	176
50s	60%	55%	55%	.	209	234
60s	71%	56%	70%	72%	.	78
70s	67%	59%	57%	64%	58%	.

Supplementary Figure S6. Gene functional analysis and pathway enrichment of DSR genes between the '20s' and '40s' age groups, with and without using 'biological sex' as covariate (p-val<0.05 and |dPSI|>0.05 on events were used to determine DSR events and genes). The Venn diagram shows the numbers of differentially spliced (DSR) genes in common and unique to the no-covariate ('no-cov') and 'biological sex'-covariate ('sex-cov') comparisons, and the tables indicate disease class, gene ontology, and pathway categories enriched in each subset of DSR genes, using a FDR<0.1 value for statistical significance. Categories in common to the comparisons are shown in black, and those unique to either of the 'no-cov' and 'sex-cov' comparisons are shown in red.

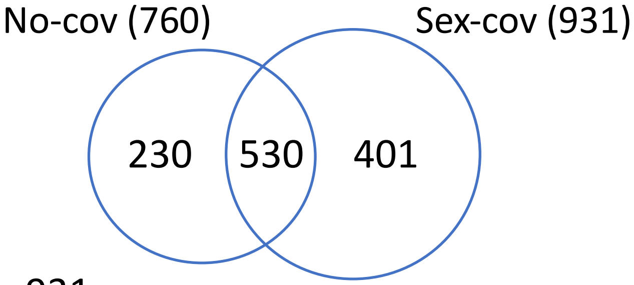

760

No-cov	
**GAD_disease_class**	Chemdependency
**GAD_disease**	Tobacco use disorder
**UP_KW_BP**	Neurogenesis
	Exocytosis
**GO_MF**	Protein binding
	Actin binding
	Cytoskeletal protein binding
**UP_KW_MF**	Kinase
	Guanine nucleotide releasing factor
	Transferase
	Actin binding
**Reactome**	Protein-protein interaction at synapses
**Wikipathways**	Splicing factor NOVA regulated synaptic proteins

230

No-cov only	

530

In common	
**GAD_disease_class**	Chemdependency
**GAD_disease**	Tobacco use disorder
**UP_KW_BP**	Neurogenesis
	Exocytosis
**GO_MF**	Protein binding
	Cytoskeletal protein binding
	Actin binding
**UP_KW_MF**	Guanine nucleotide releasing factor
	Kinase
	Actin binding
**Reactome**	Protein protein interaction at synapses
**Wikipathways**	Splicing factor NOVA regulated synaptic protein

401

Sex-cov only	
**GAD_disease_class: Chemdependency**	
**GO_MF**	RNA binding
	Protein binding
	Phospholipid binding
**Reactome**	RHOB GTPase cycle
	RAC3 GTPase cycle
	RHOC GTPAse cycle
**Biocarta**	Thrombin signalling and protease-activated receptor
**KEGG**	Endocrine and other factor-regulated calcium reabso

931

Sex-cov	
**GAD_disease_class**	Chemdependency
**GAD_disease**	Tobacco use disorder
**UP_KW_BP**	Neurogenesis
	Protein transport
	Endocytosis
	Exocytosis
**GO_MF**	Protein binding
	Cytoskeletal protein binding
	Actin binding
	Cadherin binding
	Phospholipid binding
	RNA binding
	Guanyl-nucleotide exchange factor activity
	Phosphatidylinositol-4,5-bisphosphate binding
**UP_KW_MF**	Actin binding
	Guanine nucleotide releasing factor
	Kinase
	RNA binding
	Transferase
**Reactome**	RHO GTPase cycle
	Signalling by RHO GTPases
	RHOB GTPase cycle
	RHOA GTPase cycle
	Protein protein interaction at synapses
	RAC3 GTPase cycle
	VEGFA-VEGFR2 pathway
	RHOC GTPAse cycle
	Neuronal system
	Membrane trafficking
**KEGG**	Endocytosis
	Insulin secretion
**Wikipathways**	Splicing factor NOVA regulated synaptic proteins
	EGF-EGFR signalling

Supplementary Figure S7. List of RNA binding proteins from the GO molecular function enrichment analysis, unique to the 'sex-cov' '20s-vs-40s' comparison.

Current Gene List: DSR.sexcovonly-genes
Current Background: Homo sapiens
355 DAVID IDs
45 record(s)

Download File

OFFICIAL_GENE_SYMBOL	GENE NAME	Related Genes	Species
ARL6IP4	ARF like GTPase 6 interacting protein 4(ARL6IP4)	RG	Homo sapiens
AGFG1	ArfGAP with FG repeats 1(AGFG1)	RG	Homo sapiens
CASC3	CASC3 exon junction complex subunit(CASC3)	RG	Homo sapiens
DDX39B	DExD-box helicase 39B(DDX39B)	RG	Homo sapiens
EWSR1	EWS RNA binding protein 1(EWSR1)	RG	Homo sapiens
MAZ	MYC associated zinc finger protein(MAZ)	RG	Homo sapiens
R3HDM2	R3H domain containing 2(R3HDM2)	RG	Homo sapiens
RP9	RP9 pre-mRNA splicing factor(RP9)	RG	Homo sapiens
SUPT5H	SPT5 homolog, DSIF elongation factor subunit(SUPT5H)	RG	Homo sapiens
THUMPD3	THUMP domain 3 tRNA guanosine methyltransferase(THUMPD3)	RG	Homo sapiens
ADARB1	adenosine deaminase RNA specific B1(ADARB1)	RG	Homo sapiens
ALDOA	aldolase, fructose-bisphosphate A(ALDOA)	RG	Homo sapiens
CAPRIN2	caprin family member 2(CAPRIN2)	RG	Homo sapiens
CIRBP	cold inducible RNA binding protein(CIRBP)	RG	Homo sapiens
CPEB2	cytoplasmic polyadenylation element binding protein 2(CPEB2)	RG	Homo sapiens
DNM1	dynamin 1(DNM1)	RG	Homo sapiens
FIP1L1	factor interacting with PAPOLA and CPSF1(FIP1L1)	RG	Homo sapiens
FUBP3	far upstream element binding protein 3(FUBP3)	RG	Homo sapiens
GEMIN7	gem nuclear organelle associated protein 7(GEMIN7)	RG	Homo sapiens
HNRNPDL	heterogeneous nuclear ribonucleoprotein D like(HNRNPDL)	RG	Homo sapiens
HNRNPUL1	heterogeneous nuclear ribonucleoprotein U like 1(HNRNPUL1)	RG	Homo sapiens
KIF1C	kinesin family member 1C(KIF1C)	RG	Homo sapiens
MATR3	matrin 3(MATR3)	RG	Homo sapiens
MARK2	microtubule affinity regulating kinase 2(MARK2)	RG	Homo sapiens
MYO18A	myosin XVIIIA(MYO18A)	RG	Homo sapiens
NAP1L1	nucleosome assembly protein 1 like 1(NAP1L1)	RG	Homo sapiens
PPHLN1	periphilin 1(PPHLN1)	RG	Homo sapiens
PABPC4	poly(A) binding protein cytoplasmic 4(PABPC4)	RG	Homo sapiens
PRPF3	pre-mRNA processing factor 3(PRPF3)	RG	Homo sapiens
PRPF38B	pre-mRNA processing factor 38B(PRPF38B)	RG	Homo sapiens
RRP7A	ribosomal RNA processing 7 homolog A(RRP7A)	RG	Homo sapiens
RPL14	ribosomal protein L14(RPL14)	RG	Homo sapiens
RPL35	ribosomal protein L35(RPL35)	RG	Homo sapiens
SRSF4	serine and arginine rich splicing factor 4(SRSF4)	RG	Homo sapiens
SNRPN	small nuclear ribonucleoprotein polypeptide N(SNRPN)	RG	Homo sapiens
SNHG28	small nucleolar RNA host gene 28(SNHG28)	RG	Homo sapiens
SLC25A11	solute carrier family 25 member 11(SLC25A11)	RG	Homo sapiens
SND1	staphylococcal nuclease and tudor domain containing 1(SND1)	RG	Homo sapiens
STAU1	staufen double-stranded RNA binding protein 1(STAU1)	RG	Homo sapiens
STAU2	staufen double-stranded RNA binding protein 2(STAU2)	RG	Homo sapiens
TRNT1	tRNA nucleotidyl transferase 1(TRNT1)	RG	Homo sapiens
UCHL5	ubiquitin C-terminal hydrolase L5(UCHL5)	RG	Homo sapiens
UBFD1	ubiquitin family domain containing 1(UBFD1)	RG	Homo sapiens
ZC3H13	zinc finger CCCH-type containing 13(ZC3H13)	RG	Homo sapiens
ZC3H14	zinc finger CCCH-type containing 14(ZC3H14)	RG	Homo sapiens

Figure S8. Jutils PCA plots (PC 1-5) for the male-vs-female differential splicing (DSR) analysis: without (left) and with ‘age at death’ (right) as covariate. The subgroup of samples with a distinct splicing program is shown in cyan. PC5, but not PC2-PC4, with covariate introduces more variability in the data.

(no-cov) **(age-cov)**

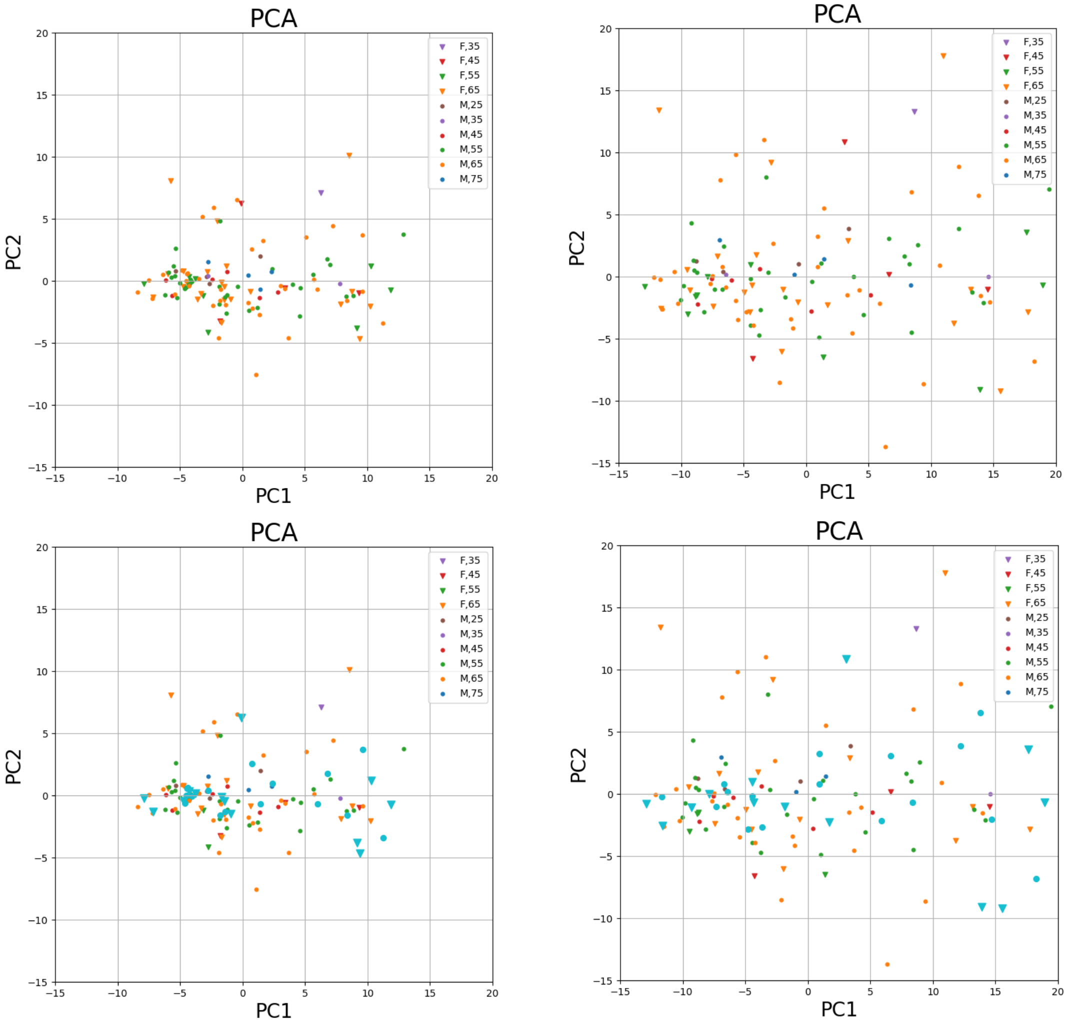

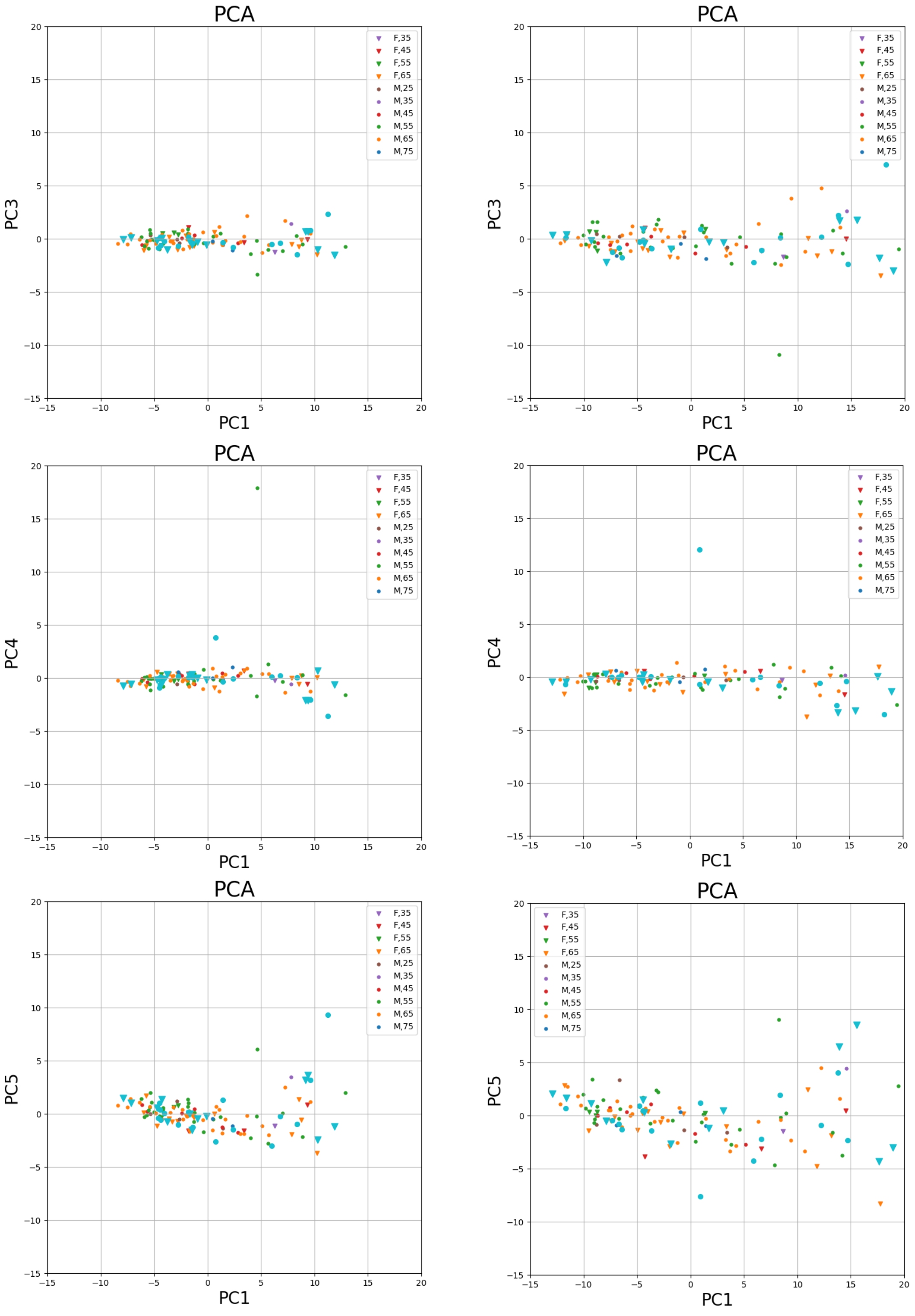
PCA
PC3
PC1
PCA
PC3
PC1
PCA
PC4
PC1
PCA
PC4
PC1
PCA
PC5
PC1
PCA
PC5
PC1
F,35
F,45
F,55
F,65
M,25
M,35
M,45
M,55
M,65
M,75

Supplementary Figure S9. Jutils heatmaps (A) and PCA plots (B) of DSA differentially spliced events (introns) between the ‘F’ and ‘M’ groups in GTEx frontal cortex data, without covariate treatment (left) and with ‘age at death’ as covariate (right). (A) Heatmaps show DSA events with p-val<0.05 and a log2 fold change of 2.0 or higher. Heatmaps were created with Jutils using the ‘cityblock’ distance metric and ‘weighted’ clustering (default). On the heatmaps, ‘F’ and ‘M’ samples form distinct clusters. The lists of genes and events are included in the Zenodo supplementary material (DOI: 10.5281/zenodo.15875405). (B) PC1, PC3 and PC5 with ‘age at death’ as covariate reduce variability in the data, whereas PC4 separates the samples into two distinguishable groups.

(A)

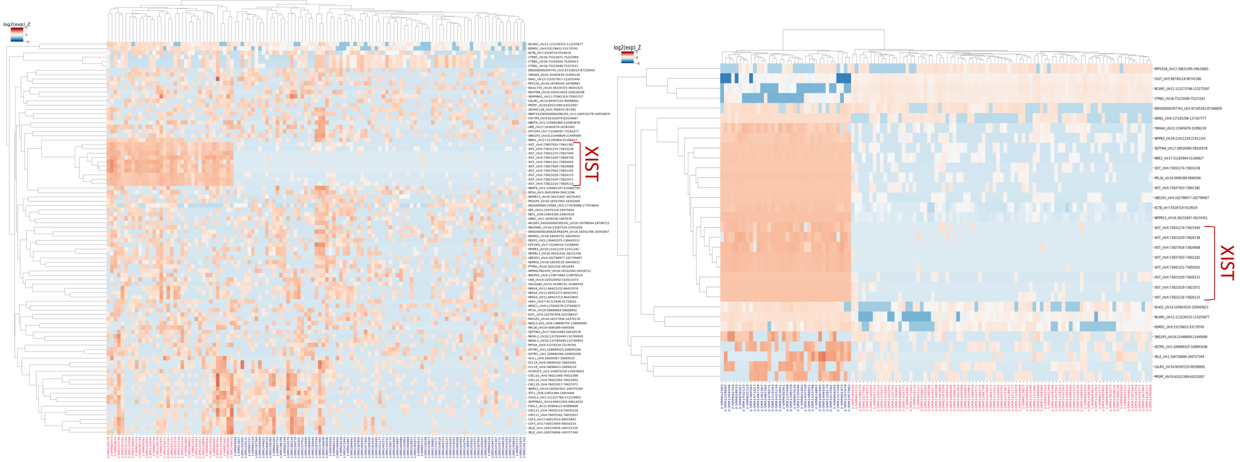

(B)

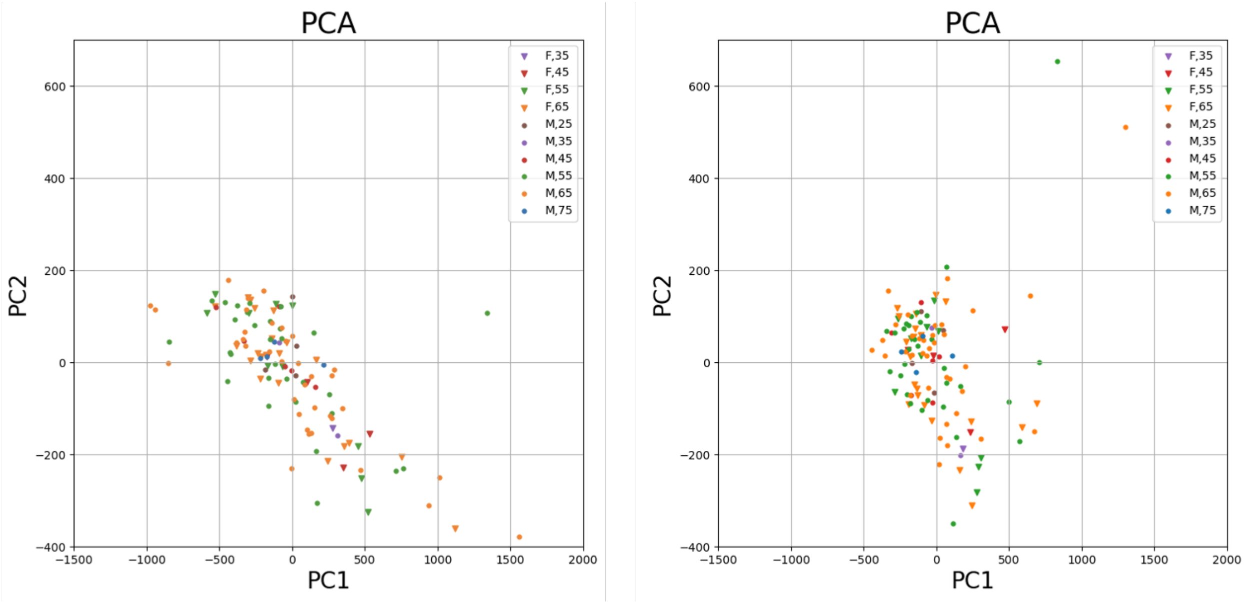

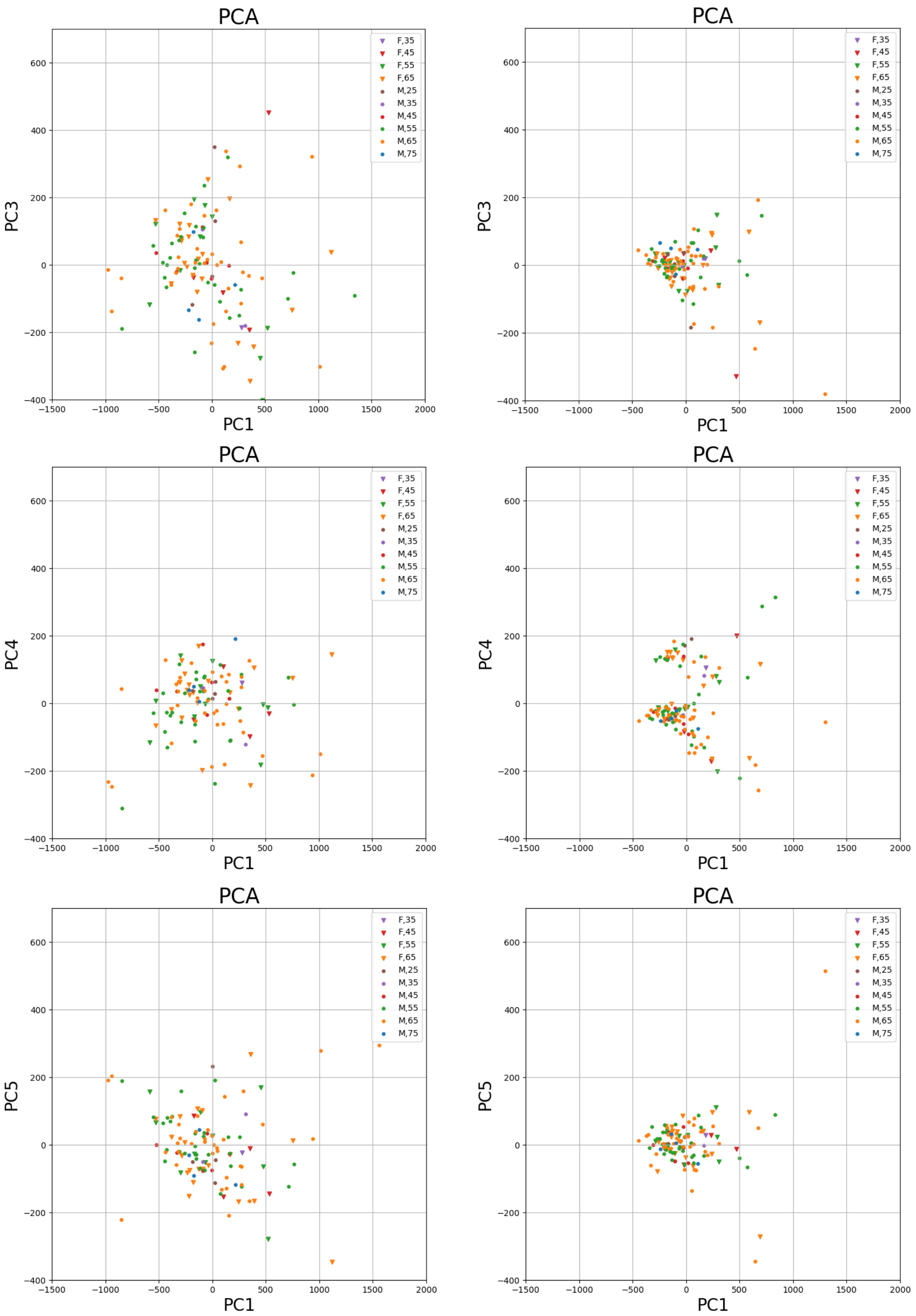
PCA
PC1
PC3
PC4
PC5
F,35
F,45
F,55
F,65
M,25
M,35
M,45
M,55
M,65
M,75
−1500
−1000
−500
0
500
1000
1500
2000
−400
−200
200
400
600

Supplementary Figure S10. Comparative gene functional analysis of DSR differentially spliced genes in the 'biological sex' ('M' versus 'F') comparison of GTEx frontal cortex samples, without ('no-cov') and with ('age-cov') accounting for 'age at death' as covariate. (A) Venn diagram of events, and genes (in parentheses). Events were filtered at p-val<0.05 and |dPSI|>=0.05, as shown in **Figure 2**. (B) Heatmap showing the top 20 enriched terms across input gene lists, colored by p-values, generated with Metascape. Associated table columns: *PATTERN* – significance in condition '1', herein 'age-cov', and/or '2', herein 'no-cov'; *GO* – enriched cluster's ID; *Category* – source (database); *Count* – cluster gene count; *Log10(p), Log10(q)* – log 10 of p-value, q-value of the comparison showing significance of differences between the conditions; and *Sex diffs. PMID* – selected literature references (PubMed identifiers) in support of male-female differences for the identified cluster/process.

(A)

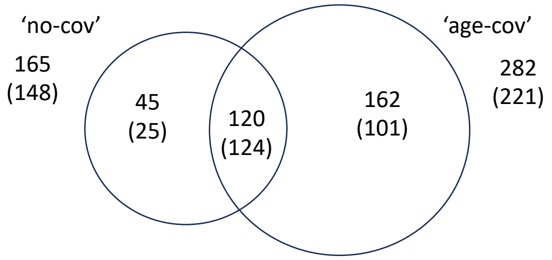

(B)

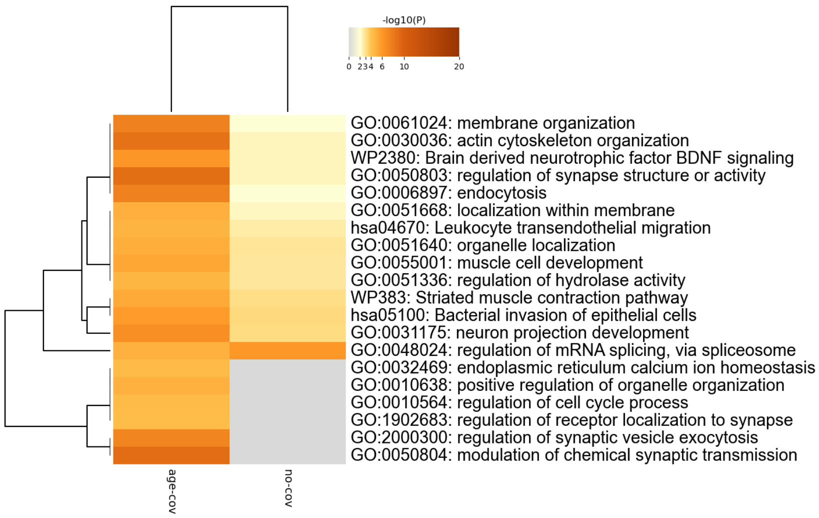

PATTERN	GO	Category	Count	Log10(p)	Log10(q)	Sex-diffs PMID
1&2	GO:0061024	GO BP	22	-7.57	-4.34	39003495
1&2	GO:0030036	GO BP	20	-8.65	-4.91	22103470
1&2	WP2380	WikiPathways	9	-6.23	-3.31	27870419
1&2	GO:0050803	GO BP	16	-8.89	-5.03	30108482
1&2	GO:0006897	GO BP	18	-7.61	-4.35	30108482
1&2	GO:0051668	GO BP	16	-5.04	-2.23	
1&2	hsa04670	KEGG	7	-4.73	-2.1	10455066
1&2	GO:0051640	GO BP	15	-5.03	-2.3	29755342
1&2	GO:0055001	GO BP	9	-5.39	-2.58	
1&2	GO:0051336	GO BP	13	-4.62	-2.01	
1&2	WP383	WikiPathways	5	-5.17	-2.43	
1&2	hsa05100	KEGG	7	-5.87	-3.01	
1&2	GO:0031175	GO BP	20	-6.7	-3.66	38764741
1&2	GO:0048024	GO BP	7	-6.03	-3.15	
1	GO:0032469	GO BP	4	-4.43	-1.86	
1	GO:0010638	GO BP	14	-4.87	-2.19	
1	GO:0010564	GO BP	17	-4.41	-1.85	30108482
1	GO:1902683	GO BP	4	-4.31	-1.8	30108482
1	GO:2000300	GO BP	7	-7.35	-4.19	30108482
1	GO:0050804	GO BP	20	-9.04	-5.03	30108482

Supplementary Figure S11. Comparative gene functional analysis of DSA differentially spliced genes in the 'biological sex' ('M' versus 'F') comparison of GTEx frontal cortex samples, without ('no-cov') and with ('age-cov') accounting for 'age at death' as covariate. (A) Venn diagram of events, and genes (in parentheses). Events were filtered at p-val<0.05 and |l2fc|>=2.0, as shown in **Supplementary Figure S8.** (B) Heatmap showing the top 20 enriched terms across input gene lists, colored by p-value, generated with Metascape. Associated table columns: *PATTERN* – significance in condition 1, herein 'age-cov', and/or '2', herein 'no-cov'; *GO* – enriched cluster's ID; *Category* – source (database); *Count* – cluster gene count*; Log10(p), Log10(q)* – log 10 of p-value, q-value of the comparison showing significance of differences between the conditions; and *Sex diffs/Aging/Other PMID* – selected literature references (PubMed identifiers) in support of male-female/aging-related/other differences for the identified cluster.

(A)

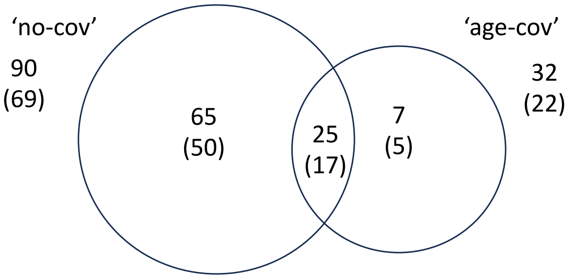

(B)

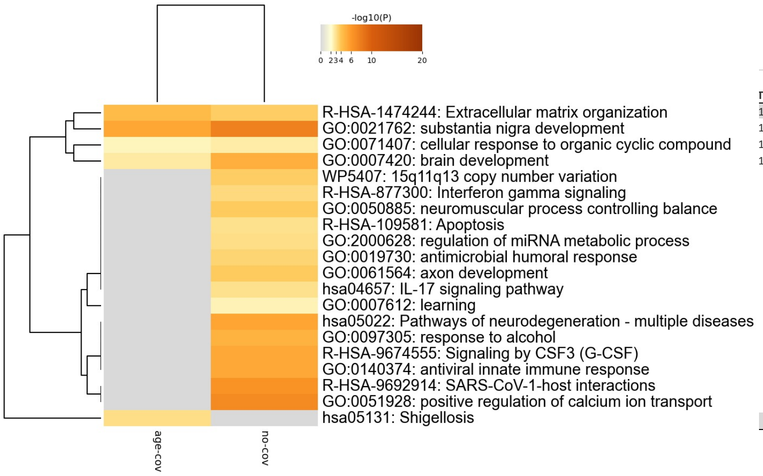

TTERN	GO	Category	Count	Log10(P)	Log10(q)	Sex-diffs PMID	Aging PMID	Other PMID
1&2	R-HSA-1474244	Reactome	6	-4.62	-1.9	35205095	38811161,25410360	
1&2	GO:0021762	GO BP	5	-7.55	-3.21	36414996	25392480	
1&2	GO:0071407	GO BP	6	-3.39	-1.17	37855285	29175000	
1&2	GO:0007420	GO BP	10	-5.59	-2.45	29977984	35847660	
2	WP5407	WikiPathways	3	-3.7	-1.41			37958807
2	R-HSA-877300	Reactome	3	-3.29	-1.15	37428916	22396896	
2	GO:0050885	GO BP	3	-3.79	-1.45		27516536	
2	R-HSA-109581	Reactome	3	-3.06	-0.99			
2	GO:2000628	GO BP	3	-3.13	-1.04			
2	GO:0019730	GO BP	5	-4.53	-1.83			
2	GO:0061564	GO BP	6	-3.78	-1.45			
2	hsa04657	KEGG	3	-3.09	-1.01		40141149	
2	GO:0007612	GO BP	5	-4.71	-1.94	37500421	16461469	
2	hsa05022	KEGG	9	-6.23	-2.77	31042697	33715827	
2	GO:0097305	GO BP	7	-5.82	-2.58			
2	R-HSA-9674555	Reactome	3	-5.27	-2.24			
2	GO:0140374	GO BP	4	-5.26	-2.24			
2	R-HSA-9692914	Reactome	5	-6.36	-2.64			
2	GO:0051928	GO BP	7	-8.35	-4.01	39737748	3829643	
1	hsa05131	KEGG	3	-3.18	-1.08			
